# Supplementary material for: Effects of mindfulness-based stress reduction on quality of life of breast cancer patient: A systematic review and meta-analysis
Source: PLoS One. 2024 Jul 19;19(7):e0306643. doi: 10.1371/journal.pone.0306643 (PMC11259293; doi:10.1371/journal.pone.0306643)
Supplement: S1 Checklist — (DOC) [file pone.0306643.s001.doc]

**PRISMA-P (Preferred Reporting Items for Systematic review and Meta-Analysis Protocols) 2015 checklist: recommended items to address in a systematic review protocol***

| Section and topic | Item No | Checklist item |
| --- | --- | --- |
| ADMINISTRATIVE INFORMATION | | |
| Title: |  |  |
| Identification | 1a | Identify the report as a protocol of a systematic review |
| Update | 1b | If the protocol is for an update of a previous systematic review, identify as such |
| Registration | 2 | If registered, provide the name of the registry (such as PROSPERO) and registration number： CRD42023459075 |
| Authors: |  |  |
| Contact | 3a | Provide name, institutional affiliation, e-mail address of all protocol authors; provide physical mailing address of corresponding author：  Xiaohui [Wang (15963369027@163.com)](mailto:Wang(15963369027@163.com))\Xinying Zhu (1920822795@qq.com)\Yu Li (ly992276043@163.com)\Tongxia Zhan (ZTX2008@126.com ) ,Department of Nursing, Weifang Medical University, Weifang, Shandong, China.  Zhicheng Dai (15666645592@163.com),Department of Clinical Medicine, Weifang Medical University, Weifang, Shandong, China.  Limin Ma (maliminwf@163.com)Department of Plastic Surgery, Weifang Medical University, Weifang, Shandong, China.  Xinghui Cui [(lcyxcxh@163.com),](mailto:(lcyxcxh@163.com),)Department of Nursing, Affiliated Hospital of Weifang Medical University, Weifang, Shandong, China. |
| Contributions | 3b | Describe contributions of protocol authors and identify the guarantor of the review:**Xiaohui Wang** (Conceptualization  \Data curation\Formal analysis\Investigation\Methodology\Writing – original draft\Writing – review & editing). **Zhicheng Dai** (Conceptualization\Data curation\Formal analysis\Methodology\Software\Writing – original draft).**Xinying Zhu** (Investigation\Methodology). Yu Li (Investigation\Methodology).**Limin Ma** (Resources\Supervision\Validation). Tongxia Zhan (Conceptualization\Methodology\Supervision\Validation) |
| Amendments | 4 | If the protocol represents an amendment of a previously completed or published protocol, identify as such and list changes; otherwise, state plan for documenting important protocol amendments:N\A |
| Support: |  |  |
| Sources | 5a | Indicate sources of financial or other support for the review |
| Sponsor | 5b | Provide name for the review funder and/or sponsor:This work was supported by the Weifang Science and Technology Development Plan college class [grant number 2021GX061](Limin Ma)、Research Fund Project of School of Nursing, Weifang Medical University [grant number 2022MS004](Tongxia Zhan) and Weifang Medical College Nursing Research Fund 2022 annual project, 2022MS003(Xinghui Cui) |
| Role of sponsor or funder | 5c | Describe roles of funder(s), sponsor(s), and/or institution(s), if any, in developing the protocol |
| INTRODUCTION | | |
| Rationale | 6 | Describe the rationale for the review in the context of what is already known:Breast cancer is the most common malignancy that occurs in women. Due to the pain caused by the disease itself and the adverse reactions in the treatment process, breast cancer patients are prone to anxiety, depression, fear of recurrence, and other negative emotions, which seriously affect the quality of life. As a systematic stress reduction therapy, mindfulness-based stress reduction is widely applied to the treatment of breast cancer patients and has been found by a growing number of studies to relieve stress, regulate mood, and improve the state. However, due to the absence of recent research and uniform outcome measures, previous studies have failed to fully explain the role of mindfulness-based stress reduction in improving the quality of life in breast cancer patients. |
| Objectives | 7 | Provide an explicit statement of the question(s) the review will address with reference to participants, interventions, comparators, and outcomes (PICO):**P:**breast cancer patient  **I****:**mindfulness-based stress reduction **C:**Usual care **O:**perceived pressure (PSS), depression, anxiety, fear of relapse (FOR), coping capacity, quality of life (QOL), sleep quality, post-traumatic growth (PTG), fatigue, pain, and emotional state. |
| METHODS | | |
| Eligibility criteria | 8 | Specify the study characteristics (such as PICO, study design, setting, time frame) and report characteristics (such as years considered, language, publication status) to be used as criteria for eligibility for the review:The population, intervention, control, and outcomes (PICO) criteria in this study were determined by the coauthors as follows: assessing the quality of life in breast cancer patients treated with MBSR compared to those receiving usual care. The extracted information included the first author's name, year of publication, country, sample size, intervention details, treatment duration, age, key elements of bias risk assessment, and outcome indicator data. The extracted results were then cross-checked. |
| Information sources | 9 | Describe all intended information sources (such as electronic databases, contact with study authors, trial registers or other grey literature sources) with planned dates of coverage:We conducted an analysis of both short-term and long-term quality of life in breast cancer patients. To identify relevant studies addressing the impact of MBSR in breast cancer patients, we conducted an integrated search of databases, including Web of Science (from 1946 to July 2023), PubMed (from 1966 to July 2023), Embase (from 1974 to July 2023), China's National Knowledge Infrastructure (from 1976 to July 2023), and the Central Cochrane Registry of Controlled Trials (from 1997 to July 2023). |
| Search strategy | 10 | Present draft of search strategy to be used for at least one electronic database, including planned limits, such that it could be repeated:PubMed |
| Study records: |  |  |
| Data management | 11a | Describe the mechanism(s) that will be used to manage records and data throughout the review:In this study, two researchers independently screened the literature and completed data extraction.Any disagreements during data extraction were resolved through discussion and adjudicated by a third senior investigator. For literature lacking original data, we attempted to contact the authors to obtain the raw data; otherwise, the studies were excluded.Data were analyzed using Revman 5.3 software. Results for continuous data are presented as mean difference (MD) and 95% confidence interval (CI). |
| Selection process | 11b | State the process that will be used for selecting studies (such as two independent reviewers) through each phase of the review (that is, screening, eligibility and inclusion in meta-analysis):In this study, two researchers independently screened the literature and completed data extraction.Any disagreements during data extraction were resolved through discussion and adjudicated by a third senior investigator. For literature lacking original data, we attempted to contact the authors to obtain the raw data; otherwise, the studies were excluded. |
| Data collection process | 11c | Describe planned method of extracting data from reports (such as piloting forms, done independently, in duplicate), any processes for obtaining and confirming data from investigators:In this study, two researchers independently screened the literature and completed data extraction.Any disagreements during data extraction were resolved through discussion and adjudicated by a third senior investigator. For literature lacking original data, we attempted to contact the authors to obtain the raw data; otherwise, the studies were excluded. |
| Data items | 12 | List and define all variables for which data will be sought (such as PICO items, funding sources), any pre-planned data assumptions and simplifications:This work was supported by the Weifang Science and Technology Development Plan college class [grant number 2021GX061]、Research Fund Project of School of Nursing, Weifang Medical University [grant number 2022MS004] and Weifang Medical College Nursing Research Fund 2022 annual project, 2022MS003 |
| Outcomes and prioritization | 13 | List and define all outcomes for which data will be sought, including prioritization of main and additional outcomes, with rationale:Primary outcome measures included perceived stress (PSS), depression, anxiety, fear of relapse (FOR), and coping ability. Secondary outcome measures included quality of life, sleep quality, post-traumatic growth (PTG), fatigue, pain, and emotional state. |
| Risk of bias in individual studies | 14 | Describe anticipated methods for assessing risk of bias of individual studies, including whether this will be done at the outcome or study level, or both; state how this information will be used in data synthesis:Data were analyzed using Revman 5.3 software. Results for continuous data are presented as mean difference (MD) and 95% confidence interval (CI). Heterogeneity among studies was assessed using the χ2 and I2 tests. A P value for the Q statistic of < 0.10 and I2 > 50% indicated significant heterogeneity, prompting the use of a random-effects model. Subgroup analysis was conducted to explore the effects of publication date, number of participants, and duration of treatment. In cases where the data from 11 articles were expressed as a median, the algorithm of Hozo et al. [31] was employed to estimate the weighted mean and standard deviation. The test for overall effects determined statistical significance by the magnitude of the p-value, considering data as statistically significant when p < 0.05. Publication bias was assessed using funnel plots. |
| Data synthesis | 15a | Describe criteria under which study data will be quantitatively synthesised:We assessed the quality of life using 11 outcome measures for comparison. Studies were deemed eligible if they included one of the following outcome measures: perceived pressure (PSS), depression, anxiety, fear of relapse (FOR), coping capacity, quality of life (QOL), sleep quality, post-traumatic growth (PTG), fatigue, pain, and emotional state. In the included studies, mindfulness-based stress reduction was implemented in the experimental group, while usual care was provided in the control group. We excluded meeting notes and abstracts lacking complete RCTs, as well as studies with incomplete data. In cases of replicated published studies, we selected articles with available data and the most recent results. To ensure a comprehensive search for data, we also reviewed the references of the included studies. |
|  | 15b | If data are appropriate for quantitative synthesis, describe planned summary measures, methods of handling data and methods of combining data from studies, including any planned exploration of consistency (such as I^2^, Kendall’s τ): The test for overall effects determined statistical significance by the magnitude of the p-value, considering data as statistically significant when p < 0.05. Publication bias was assessed using funnel plots. |
|  | 15c | Describe any proposed additional analyses (such as sensitivity or subgroup analyses, meta-regression):subgroup analyses |
|  | 15d | If quantitative synthesis is not appropriate, describe the type of summary planned |
| Meta-bias(es) | 16 | Specify any planned assessment of meta-bias(es) (such as publication bias across studies, selective reporting within studies):All included studies underwent quality assessment using the JADAD scale, which evaluates four aspects: random sequence generation, random hiding, blinding, and exit. The tool assigns ratings on a scale of 1 to 7, with scores of 1 to 3 considered low-quality literature and scores of 4 to 7 considered high-quality literature. To assess the risk of bias, we utilized the Cochrane Bias tool for RCTs, which includes seven assessments: sequence generation, assignment hiding, subject blindness, outcome evaluators, exit and loss of follow-up, incomplete outcome data, and selective outcome reporting. The included literature was categorized as low risk, high risk, or unclear. Two independent reviewers conducted the assessment, and any discrepancies were resolved through negotiation with a third researcher. |
| Confidence in cumulative evidence | 17 | Describe how the strength of the body of evidence will be assessed (such as GRADE): JADAD scale |

*** It is strongly recommended that this checklist be read in conjunction with the PRISMA-P Explanation and Elaboration (cite when available) for important clarification on the items. Amendments to a review protocol should be tracked and dated. The copyright for PRISMA-P (including checklist) is held by the PRISMA-P Group and is distributed under a Creative Commons Attribution Licence 4.0.**

*From: Shamseer L, Moher D, Clarke M, Ghersi D, Liberati A, Petticrew M, Shekelle P, Stewart L, PRISMA-P Group. Preferred reporting items for systematic review and meta-analysis protocols (PRISMA-P) 2015: elaboration and explanation. BMJ. 2015 Jan 2;349(jan02 1):g7647.*
