# Supplementary figures and images for: Effects of mindfulness-based stress reduction on quality of life of breast cancer patient: A systematic review and meta-analysis
Source: PLoS One. 2024 Jul 19;19(7):e0306643. doi: 10.1371/journal.pone.0306643 (PMC11259293; doi:10.1371/journal.pone.0306643)

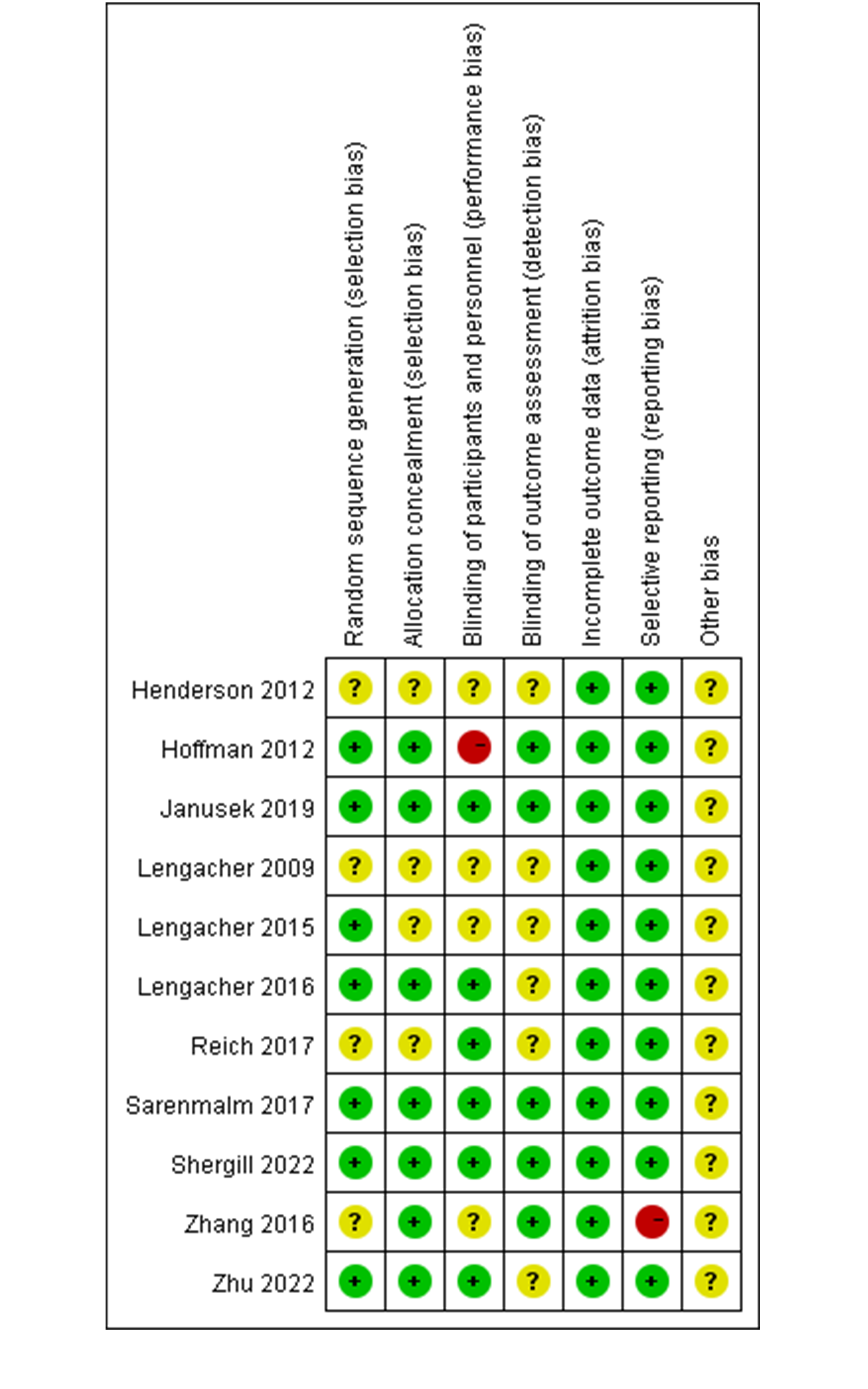

Supplement: S1 Fig — (TIF) [file pone.0306643.s002.tif]

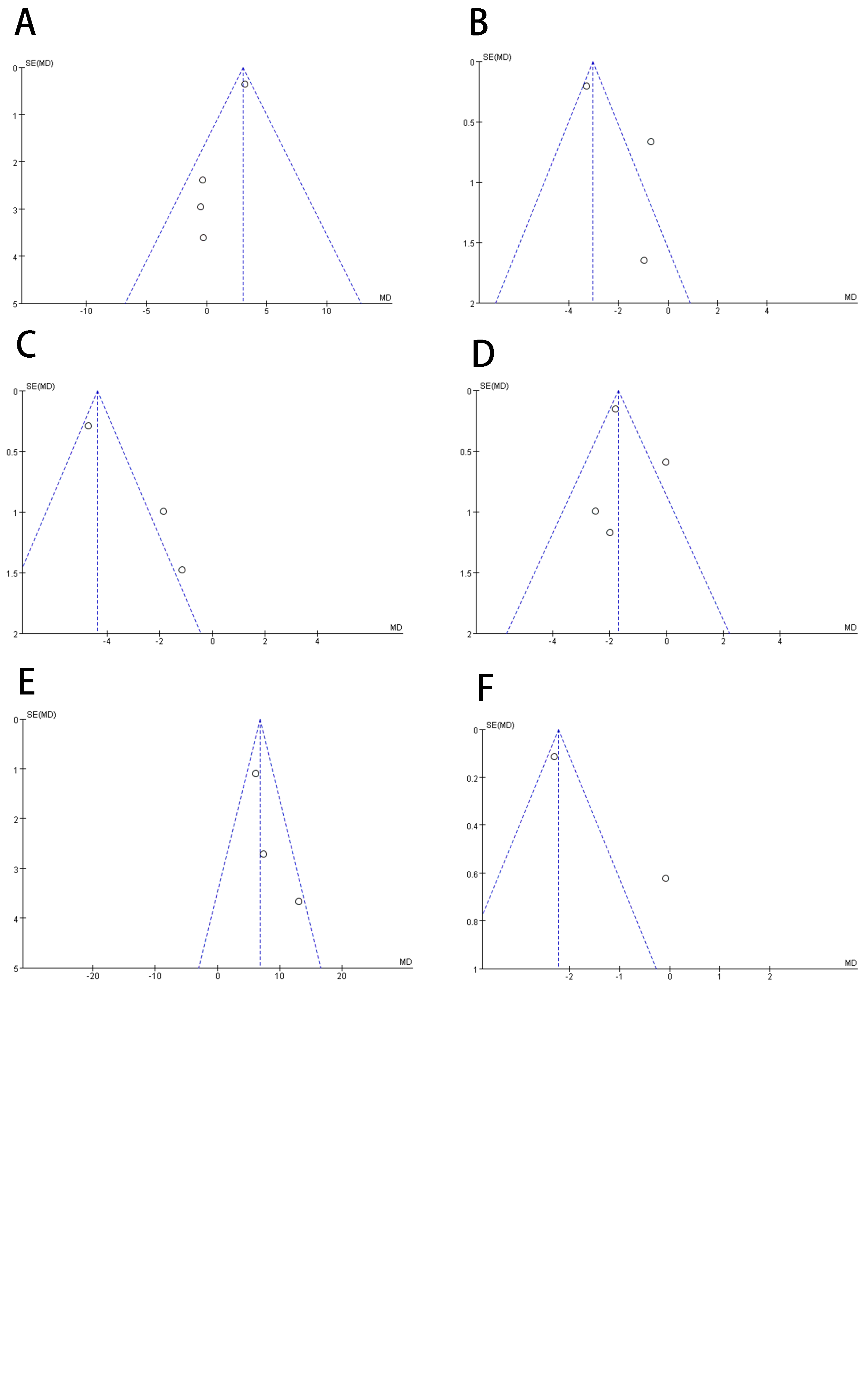

Supplement: S2 Fig — (A) HQOL(B) Depression (C) Anxiety (D) Perceived Pressure (E) Personal Growth (F) Fear of relapse. (TIF) [file pone.0306643.s003.tif]

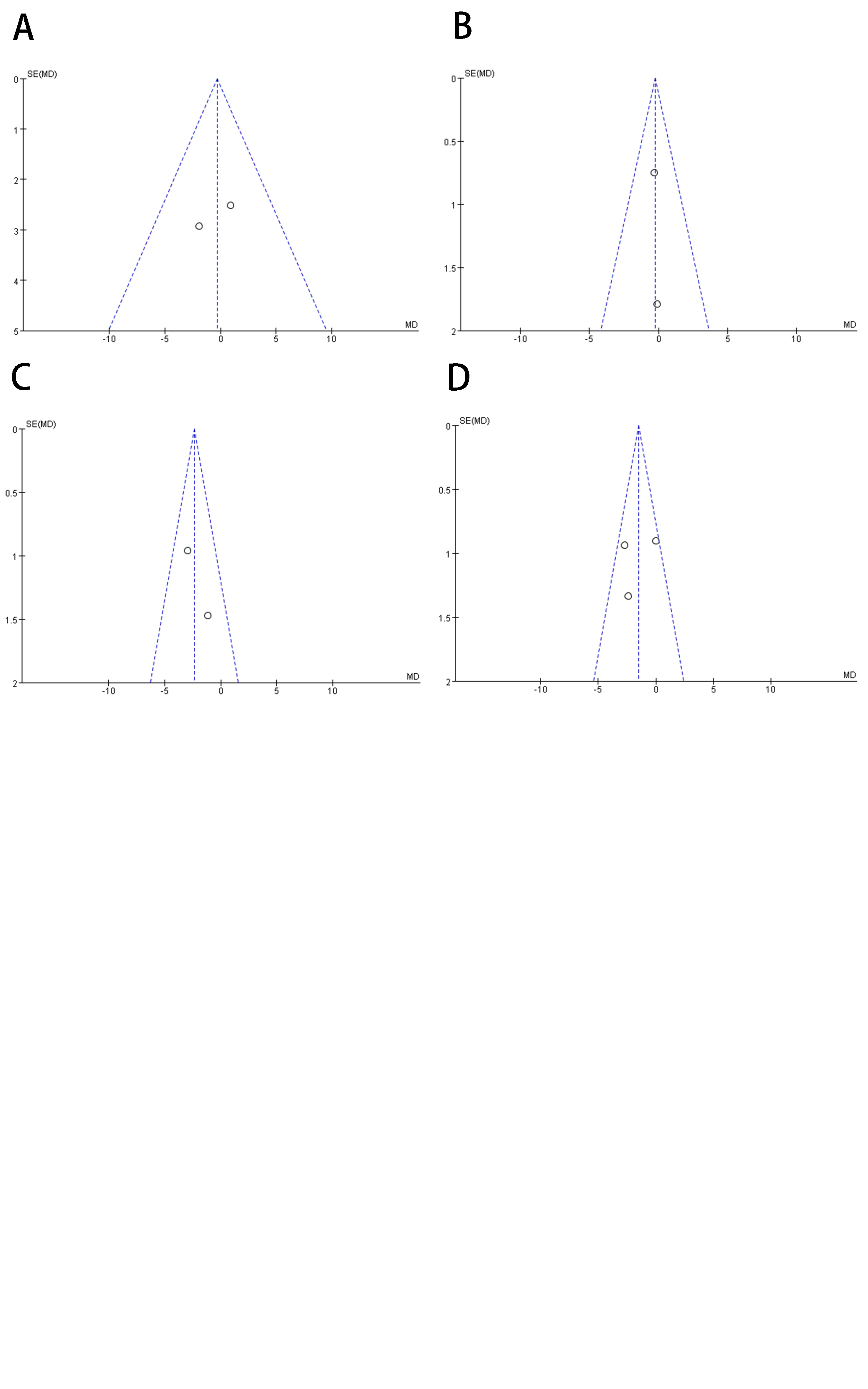

Supplement: S3 Fig — (A)HQOL(B) Depression (C) Anxiety (D) Perceived Pressure. (TIF) [file pone.0306643.s004.tif]

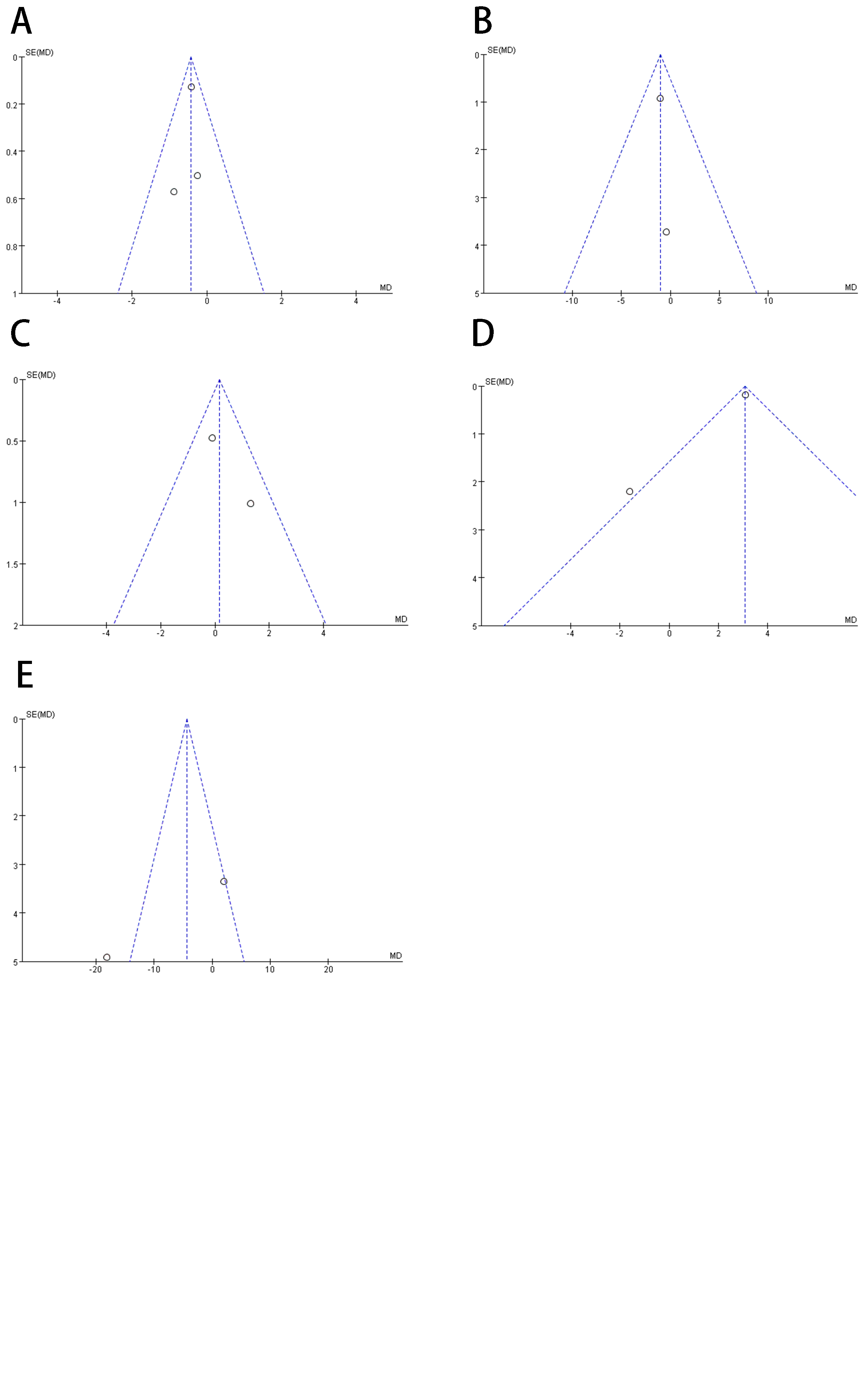

Supplement: S4 Fig — (A) Sleep Quality (B) Fatigue (C) Pain (D) Coping Capacity (E) Emotional State. (TIF) [file pone.0306643.s005.tif]

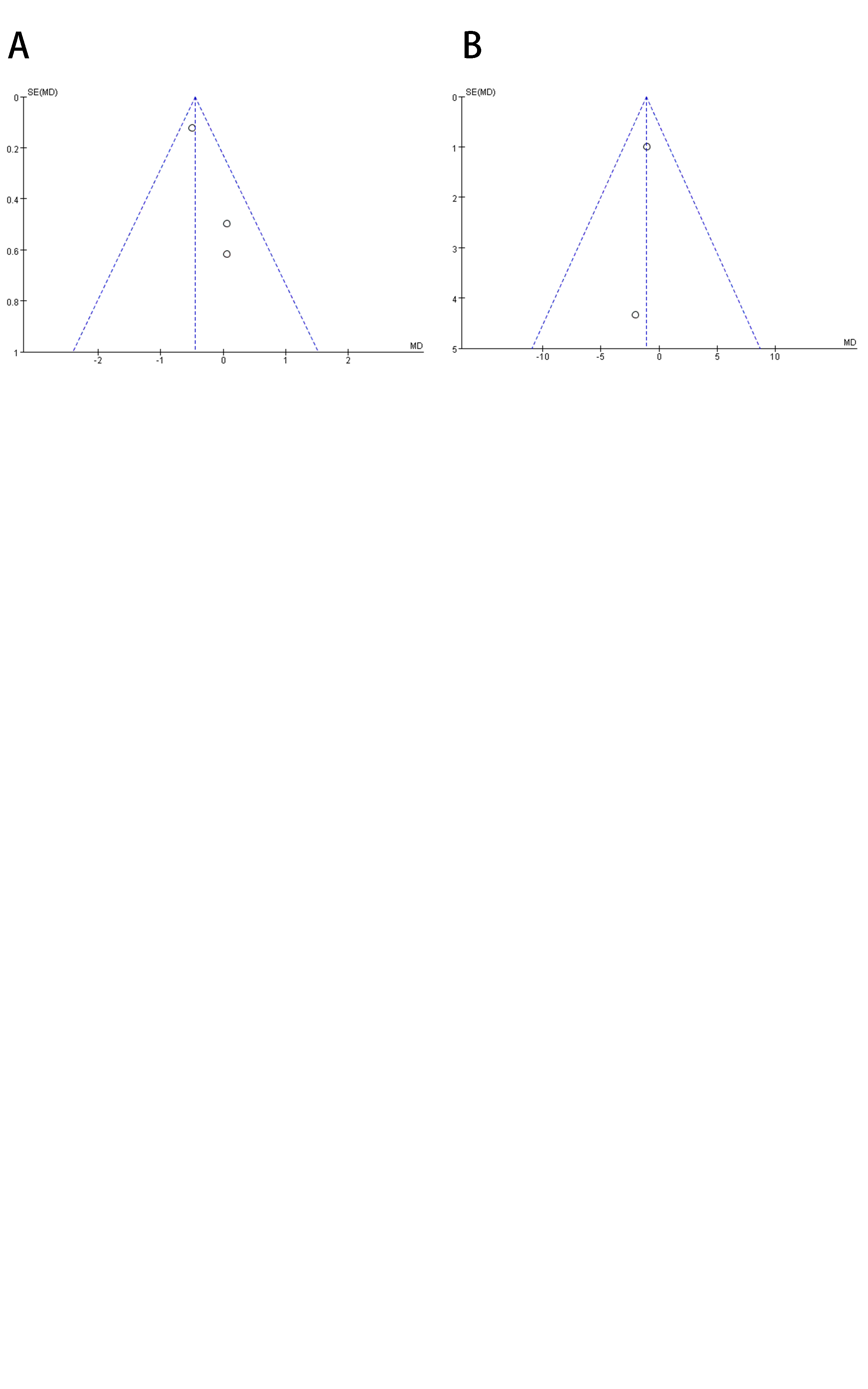

Supplement: S5 Fig — (A) Sleep Quality (B) Fatigue. (TIF) [file pone.0306643.s006.tif]
