# Supplementary material for: Effects of mindfulness-based stress reduction on quality of life of breast cancer patient: A systematic review and meta-analysis
Source: PLoS One. 2024 Jul 19;19(7):e0306643. doi: 10.1371/journal.pone.0306643 (PMC11259293; doi:10.1371/journal.pone.0306643)
Supplement: S1 Raw data — (DOCX) [file pone.0306643.s007.docx]

1.Raw data before and after intervention

| Outcome indicators | Study | Intervention group |  |  | Control group |  |  |
| --- | --- | --- | --- | --- | --- | --- | --- |
|  |  | Mean | SD | Total | Mean | SD | Total |
| Sleep Quality | Janusek2019 | 6.48 | 3.12 | 70 | 7.36 | 3.55 | 67 |
|  | Lengacher2015 | 7.97 | 5.06 | 38 | 8.39 | 3.63 | 41 |
|  | Reich2017 | 7.08 | 4.42 | 150 | 7.02 | 4.12 | 145 |
| perceived stress(PSS) | Janusek 2019 | 13.66 | 6.79 | 70 | 15.66 | 6.87 | 67 |
|  | Lengacher 2009 | 12.6 | 0.72 | 40 | 14.4 | 0.69 | 42 |
|  | Reich 2017 | 12.9 | 7.75 | 158 | 12.89 | 8.2 | 154 |
|  | Zhang 2016 | 31.54 | 3.75 | 28 | 34.27 | 3.36 | 30 |
| Depression | Janusek 2019 | 10.46 | 10.4 | 70 | 11.42 | 8.8 | 67 |
|  | Lengacher 2009 | 6.3 | 0.95 | 40 | 9.6 | 0.92 | 42 |
|  | Lengacher 2016 | 8.66 | 6.26 | 155 | 8.95 | 6.8 | 148 |
|  | Reich 2017 | 8.66 | 6.26 | 155 | 8.95 | 6.8 | 148 |
| Anxiety | Lengacher 2009 | 28.3 | 1.32 | 40 | 33 | 1.31 | 42 |
|  | Lengacher 2016 | 31.82 | 12.1 | 155 | 32.99 | 13.4 | 148 |
|  | Reich 2017 | 31.82 | 12.1 | 155 | 32.99 | 13.4 | 148 |
|  | Zhang 2016 | 40.07 | 3.2 | 28 | 43 | 4.09 | 30 |
| quality of life | Lengacher 2009 | 51.59 | 1.16 | 40 | 48.4 | 2.02 | 42 |
|  | Lengacher 2016 | 68.76 | 21.5 | 153 | 67.85 | 21.99 | 146 |
|  | Reich 2017 | 71.33 | 19.41 | 153 | 72.71 | 19.13 | 146 |
|  | Sarenmalm 2017 | 74.1 | 17.1 | 62 | 74.4 | 20.7 | 52 |
| post-traumatic growth (PTG) | Sarenmalm 2017 | 64.65 | 17.7 | 62 | 51.57 | 20.8 | 52 |
|  | Zhang 2016 | 71.5 | 4.5 | 28 | 63.33 | 3.39 | 30 |
|  | Zhu 2022 | 66.12 | 13.136 | 50 | 58.804 | 14.112 | 51 |
| Fatigue | Janusek 2019 | 9.65 | 23.54 | 70 | 10.09 | 19.98 | 67 |
|  | Lengacher 2016 | 12.2 | 8.56 | 152 | 13.27 | 8.71 | 147 |
|  | Reich 2017 | 12.2 | 8.56 | 152 | 13.27 | 8.71 | 147 |
| Pain | Lengacher 2016 | 8.46 | 9.41 | 153 | 8.66 | 8.4 | 155 |
|  | Reich 2017 | 8.46 | 9.41 | 161 | 8.66 | 8.4 | 155 |
|  | Shergill 2022 | 3.49 | 2.09 | 49 | 3.58 | 2.61 | 49 |
| fear of relapse（FOR） | Lengacher 2009 | 9.3 | 0.53 | 40 | 11.6 | 0.51 | 42 |
|  | Lengacher 2016 | 9.25 | 5.28 | 161 | 9.61 | 5.77 | 155 |
| coping ability | Henderson 2012 | 46.8 | 1 | 53 | 43.7 | 1 | 58 |
|  | Sarenmalm 2017 | 67.7 | 12 | 62 | 69.3 | 11.5 | 52 |
| emotional state | Hoffman 2012 | 30.02 | 31.6 | 103 | 48.08 | 39.89 | 111 |
|  | Shergill 2022 | 62.88 | 16.67 | 49 | 60.92 | 16.46 | 49 |

2.Raw data before and after follow-up

| post-follow-up outcome indicators | Study | Intervention group |  |  | Control group |  |  |
| --- | --- | --- | --- | --- | --- | --- | --- |
|  |  | Mean | SD | Total | Mean | SD | Total |
| Sleep Quality | Janusek 2019 | 6.94 | 3.26 | 63 | 6.88 | 3.58 | 61 |
|  | Lengacher 2015 | 6.91 | 0.56 | 38 | 7.41 | 0.54 | 41 |
|  | Reich 2017 | 7.08 | 4.42 | 150 | 7.02 | 4.12 | 145 |
| perceived stress(PSS) | Janusek 2019 | 13.7 | 7.1 | 63 | 16.09 | 7.76 | 61 |
|  | Reich 2017 | 12.9 | 7.75 | 158 | 12.89 | 8.2 | 154 |
|  | Zhang 2016 | 31.54 | 3.75 | 28 | 34.27 | 3.36 | 30 |
| Depression | Janusek 2019 | 10.57 | 9.5 | 63 | 10.69 | 10.38 | 61 |
|  | Lengacher 2016 | 8.66 | 6.26 | 155 | 8.95 | 6.8 | 148 |
| Anxiety | Lengacher 2016 | 31.82 | 12.1 | 155 | 32.99 | 13.4 | 148 |
|  | Zhang 2016 | 40.07 | 3.2 | 28 | 43 | 4.09 | 30 |
| quality of life | Lengacher 2016 | 68.76 | 21.5 | 153 | 67.85 | 21.99 | 146 |
|  | Reich 2017 | 68.43 | 27.76 | 153 | 70.36 | 22.7 | 146 |
| Fatigue | Janusek 2019 | 8.28 | 22.46 | 63 | 10.33 | 25.58 | 61 |
|  | Lengacher 2016 | 12.2 | 8.56 | 152 | 13.27 | 8.71 | 147 |
