# Supplementary material for: Effects of mindfulness-based stress reduction on quality of life of breast cancer patient: A systematic review and meta-analysis
Source: PLoS One. 2024 Jul 19;19(7):e0306643. doi: 10.1371/journal.pone.0306643 (PMC11259293; doi:10.1371/journal.pone.0306643)
Supplement: Attachment — Submitted filename: Response letter.docx [file pone.0306643.s009.docx]

Dear editor,

Thank you for your letter and for the reviewers’ valuable comments concerning our manuscript entitled “Effects of mindfulness-based stress reduction on quality of life of breast cancer patient: A systematic review and meta-analysis”. These comments are of great help to the revision and improvement of our article, and help to improve the quality of the article. All authors have carefully studied the comments and made revisions with the hope of publication in PLOS ONE. Additionally, We modified the format of the manuscript according to PLOS ONE's style requirements. The reference section has also been revised. It should be noted that the name of our school was changed from "Weifang Medical College" to "Shandong Second Medical University" last December, so I changed the unit in the article to "Shandong Second Medical University".

A list of our responses to specific recommendations follows. If there are any other modifications we could make, we would like very much to modify them and we really appreciate your help.

Yours sincerely,

Xiaohui Wang

Response to Reviewers:

Thank you very much for your revision and comments in the article. The following are my answers to your questions.

**1. What do you mean by this? This is sexist langauge.the use of words such as delicate and sensitive are inappropriate for a scientific paper. I would omit this line.**

***Response:*** Thank you for your comment. As per your suggestion, we change “Malignant tumors have a significant psychological impact on patients, and women are often more delicate and sensitive than men” to “Malignant tumor has significant effect on female mental health”(Page 4, line 84).

**2.What do you mean by this?? Emotions do not operate as pathogens, You can state that one's emotional state can influence the affective states of others.**

***Response:*** Thank you for your comment. We change “Emotions are contagious” to “One person's emotional state affects the emotional state of others”(Page 4, line 87 ).

**3. Do not use metaphors such as casting a dark shadow. This is unscientific.**

***Response:*** Thank you for your valuable suggestion. We Change “Negative and depressed emotions not only cast a shadow on the entire family” to “Negativity and depression don't just have a negative impact on the entire family” (Page 4, line 90).

**4.Some language problems in the article.**

***Response:*** Thank you for your comment. We have made all modifications according to the annotations in the article.

**5.You should state prior to 2018.**

***Response:*** Thank you for your help and valuable advice. We have moved the position of this paragraph to line 113 before "Although"

**6.The correct** **method to write author and year is Henderson (2012). do this for the other authors.**

***Response:*** Thank you for your comment. We have adopted your comments and revised the method to write.

**7. Do not use the word ''usual'. change this word to standard in the other occasions where it is used.**

***Response:*** Thank you for your valuable advice. We have revised all the usual care in this article to standard care.

8. **Use the non-abbreviated name at the start of a sentence.**

***Response:*** Thank you for your valuable advice. We have made modifications according to your suggestion (Page 25, line 399).
