# Supplementary material for: Effects of mindfulness-based stress reduction on quality of life of breast cancer patient: A systematic review and meta-analysis
Source: PLoS One. 2024 Jul 19;19(7):e0306643. doi: 10.1371/journal.pone.0306643 (PMC11259293; doi:10.1371/journal.pone.0306643)
Supplement: Attachment — Submitted filename: Effects of mindfulness-Reviewer Comments.pdf [file pone.0306643.s008.pdf]

PLOS ONE

Effects of mindfulness-based stress reduction on quality of life of breast cancer patient: A meta-analysis

--Manuscript Draft--

**Manuscript Number:**	PONE-D-23-40328
**Article Type:**	Research Article
**Full Title:**	Effects of mindfulness-based stress reduction on quality of life of breast cancer patient: A meta-analysis
**Short Title:**	Effects of mindfulness-based stress reduction on quality of life of breast cancer patient
**Corresponding Author:**	Tongxia Zhan<br>Weifang Medical University<br>Weifang, CHINA
**Keywords:**	Mindfulness-based stress reduction; breast cancer; MBSR; quality of life; Negative Emotions; Usual Care.
**Abstract:**	A bstract: Backg r ound: Breast cancer is the most common malignancy that occurs in women, due to the pain caused by disease itself and the adverse reactions in the treatment process, breast cancer patients are prone to anxiety, depression, fear of recurrence and other negative emotions, which seriously affect the quality of life. As a systematic stress reduction method, mindfulness-based stress reduction (MBSR) is widely applied to the treatment of breast cancer patients and has been found by a growing number of studies to relieve stress, regulate mood and improve state. However, due to the absence of recent researches and uniform outcome measures, previous studies have failed to fully explain the role of mindfulness-based stress reduction in improving quality of life in breast cancer patients.<br>Objective: We conducted a systematic review and meta-analysis to evaluate and compare the effects of mindfulness-based stress reduction therapy (MBSR) and usual care on quality of life and psychological status in breast cancer patients. Methods: We searched PubMed, Web of Science, Embase, China's National Knowledge Infrastructure, and the Cochrane Central Registry of Controlled Trials up to July 2023 to identify candidate randomized clinical trials, which addressing the values of MBSR in breast cancer patients. The endpoint was to assess the improvement on quality of life in breast cancer patients treated with MBSR using a meta-analysis and to compare them with usual care. Result: A total of 1644 patients participated in 11 randomized controlled trials. The results of meta-analysis showed that mindfulness-based stress reduction therapy can be significant reduce the negative emotions such as PSS (MD ＝ -1.46，95%CI = -2.53 to -0.38，p = 0.03), depression (MD = -1.84，95%CI = -3.99 to -0.30，p = 0.0004), anxiety (MD = -2.81，95%CI = -5.31 to -0.32，p = 0.002) and fear of recurrence (MD = -1.27, 95%CI = -3.44 to 0.90，p = 0.0004). Mindfulness-based stress reduction therapy also has certain advantages in improving the coping ability (MD = 1.26, 95%CI = -3.23 to5.76, p = 0.03) and the emotional state (MD = -7.73, 95%CI = -27.34 to 11.88, p = 0.0007) of patients with breast cancer.<br>Conclusion: Our analyses support that compared with usual care, mindfulness-based stress reduction therapy can significantly improve patients' coping ability, reduce adverse emotions such as depression, anxiety, perceived stress and fear of recurrence, and improve patients' emotional state. These results have to be confirmed in a larger cohort. Nevertheless, our study might help policymakers and stakeholders evaluate the clinical and social impact of the MBSR in breast cancer patients.
**Order of Authors:**	Xiaohui Wang
	Zhicheng Dai
	Xinying Zhu
	Yu Li
	Limin Ma
	Xinghui Cui

Powered by Editorial Manager® and ProduXion Manager® from Aries Systems Corporation

	Tongxia Zhan
**Additional Information:**	
**Question**	**Response**
**Financial Disclosure**<br><br>Enter a financial disclosure statement that describes the sources of funding for the work included in this submission. Review the submission guidelines for detailed requirements. View published research articles from *PLOS ONE* for specific examples.<br><br>This statement is required for submission and **will appear in the published article** if the submission is accepted. Please make sure it is accurate.<br><br>**Funded studies**<br>Enter a statement with the following details:<br>• Initials of the authors who received each award<br>• Grant numbers awarded to each author<br>• The full name of each funder<br>• URL of each funder website<br>• Did the sponsors or funders play any role in the study design, data collection and analysis, decision to publish, or preparation of the manuscript?<br><br>Did you receive funding for this work?	The author(s) received no specific funding for this work.
**Competing Interests**<br><br>Use the instructions below to enter a competing interest statement for this submission. On behalf of all authors, disclose any competing interests that could be perceived to bias this work—acknowledging all financial support and any other relevant financial or non-financial competing interests.<br><br>This statement is required for submission and **will appear in the published article** if the submission is accepted. Please make sure it is accurate and that any funding	The authors have declared that no competing interests exist.

sources listed in your Funding Information later in the submission form are also declared in your Financial Disclosure statement.<br><br>View published research articles from *PLOS ONE* for specific examples.<br><br>**NO authors have competing interests**<br><br>Enter: *The authors have declared that no competing interests exist.*<br><br>**Authors with competing interests**<br><br>Enter competing interest details beginning with this statement:<br><br>*I have read the journal's policy and the authors of this manuscript have the following competing interests: [insert competing interests here]*<br><br>* typeset	
**Ethics Statement**<br><br>Enter an ethics statement for this submission. This statement is required if the study involved:<br><br>• Human participants<br>• Human specimens or tissue<br>• Vertebrate animals or cephalopods<br>• Vertebrate embryos or tissues<br>• Field research<br><br>Write "N/A" if the submission does not require an ethics statement.<br><br>General guidance is provided below. Consult the submission guidelines for detailed instructions. **Make sure that all information entered here is included in the Methods section of the manuscript.**	N/A

**Format for specific study types**<br><br>**Human Subject Research (involving human participants and/or tissue)**<br>• Give the name of the institutional review board or ethics committee that approved the study<br>• Include the approval number and/or a statement indicating approval of this research<br>• Indicate the form of consent obtained (written/oral) or the reason that consent was not obtained (e.g. the data were analyzed anonymously)<br><br>**Animal Research (involving vertebrate animals, embryos or tissues)**<br>• Provide the name of the Institutional Animal Care and Use Committee (IACUC) or other relevant ethics board that reviewed the study protocol, and indicate whether they approved this research or granted a formal waiver of ethical approval<br>• Include an approval number if one was obtained<br>• If the study involved *non-human primates*, add *additional details* about animal welfare and steps taken to ameliorate suffering<br>• If anesthesia, euthanasia, or any kind of animal sacrifice is part of the study, include briefly which substances and/or methods were applied<br><br>**Field Research**<br><br>Include the following details if this study involves the collection of plant, animal, or other materials from a natural setting:<br>• Field permit number<br>• Name of the institution or relevant body that granted permission	
**Data Availability**<br><br>Authors are required to make all data underlying the findings described fully available, without restriction, and from the time of publication. PLOS allows rare exceptions to address legal and ethical concerns. See the PLOS Data Policy and FAQ for detailed information.	Yes - all data are fully available without restriction

A Data Availability Statement describing where the data can be found is required at submission. Your answers to this question constitute the Data Availability Statement and **will be published in the article**, if accepted.<br><br>**Important:** Stating 'data available on request from the author' is not sufficient. If your data are only available upon request, select 'No' for the first question and explain your exceptional situation in the text box.<br><br>Do the authors confirm that all data underlying the findings described in their manuscript are fully available without restriction?	
**Describe where the data may be found in full sentences. If you are copying our sample text, replace any instances of XXX with the appropriate details.**<br><br>• If the data are **held or will be held in a public repository**, include URLs, accession numbers or DOIs. If this information will only be available after acceptance, indicate this by ticking the box below. For example: *All XXX files are available from the XXX database (accession number(s) XXX, XXX.).*<br>• If the data are all contained **within the manuscript and/or Supporting Information files**, enter the following: *All relevant data are within the manuscript and its Supporting Information files.*<br>• If neither of these applies but you are able to provide **details of access elsewhere**, with or without limitations, please do so. For example:<br><br>*Data cannot be shared publicly because of [XXX]. Data are available from the XXX Institutional Data Access / Ethics Committee (contact via XXX) for researchers who meet the criteria for access to confidential data.*<br><br>*The data underlying the results presented in the study are available from (include the name of the third party*	All relevant data are within the manuscript and its Supporting Information files.

and contact information or URL).<br>• This text is appropriate if the data are owned by a third party and authors do not have permission to share the data.<br><br>* typeset	
Additional data availability information:	

Click here to access/download;Manuscript;Final manuscript.docx

Effects of mindfulness-based stress reduction on quality of life of breast cancer patient: A systematic review and meta-analysis

Xiaohui Wang[1¶], Zhicheng Dai[2¶], Xinying Zhu[1], Yu Li[1], Limin Ma[3], Xinghui Cui[4], Tongxia Zhan[1]*

[1]Department of Nursing, Weifang Medical University, Weifang, Shandong, China

[2]Department of Clinical Medicine, Weifang Medical University, Weifang, Shandong, China

[3]Department of Plastic Surgery, Weifang Medical University, Weifang, Shandong, China

[4]Department of Nursing, Affiliated Hospital of Weifang Medical University, Weifang, Shandong, China

*Corresponding author

E-mail: ZTX2008@126.com (TZ)

[¶]These authors contributed equally to this work.

Abstract

Background: Breast cancer is the most common malignancy that occurs in women. Due to the pain caused by the disease itself and the adverse reactions in the treatment process, breast cancer patients are prone to anxiety, depression, fear of recurrence, and other negative emotions, which seriously affect the quality of life. As a systematic stress reduction therapy, mindfulness-based stress reduction is widely applied to the treatment of breast cancer patients and has been found by a growing number of studies to relieve stress, regulate mood, and improve the state. However, due to the absence of recent research and uniform outcome measures, previous studies have failed to fully explain the role of mindfulness-based stress reduction in improving the quality of life in breast cancer patients.

Objective: We conducted a systematic review and meta-analysis to evaluate and compare the effects of mindfulness-based stress reduction therapy and usual care on the quality of life and psychological status of breast cancer patients.

Methods: We searched PubMed, Web of Science, Embase, China's National Knowledge Infrastructure and the Cochrane Central Registry of Controlled Trials up to July 2023 to identify candidate randomized clinical trials addressing the values of mindfulness-based stress reduction in breast cancer patients.

Results: A total of 1644 patients participated in 11 randomized controlled trials. The results of the meta-analysis showed that mindfulness-based stress reduction therapy can significantly reduce negative emotions such as perceived stress (MD = -1.46, 95%CI = -2.53 to -0.38, p = 0.03), depression (MD =-1.84, 95%CI = -3.99 to -0.30, p

= 0.0004), anxiety (MD = -2.81, 95%CI = -5.31 to -0.32, p = 0.002), and fear of recurrence (MD = -1.27, 95%CI = -3.44 to 0.90， p = 0.0004). Mindfulness-based stress reduction therapy also has certain advantages in improving the coping ability (MD = 1.26, 95%CI = -3.23 to5.76, p = 0.03) and the emotional state (MD = -7.73, 95%CI = -27.34 to 11.88, p = 0.0007) of patients with breast cancer.

Conclusion: Our analyses support that, compared with usual care, mindfulness-based stress reduction therapy can significantly improve patients' coping ability, reduce adverse emotions and improve patients' emotional states.

Keywords: Mindfulness-based stress reduction; Breast cancer; MBSR; Quality of life; Negative Emotions; Usual Care.

1. Introduction

Breast cancer is the most commonly diagnosed cancer type, with approximately 2.1 million new cases every year, making it the leading cause of death in women worldwide[1]. Early-stage breast cancer is considered curable[2]. At present, the most common treatment for breast cancer is surgical treatment, including breast-conserving surgery and sentinel lymph node biopsy. In addition to surgery, the main treatment methods for breast cancer include neoadjuvant chemotherapy, targeted therapy, endocrine therapy, and radiotherapy. Side effects can occur to varying degrees during treatment, such as the direct toxic effect of breast cancer treatment on the heart[3]. Other effects include changes in body and body image, arm diseases (lymphedema, axillary syndrome), and functional changes[4]. These adverse reactions can lead to a

range of psychological problems in breast cancer patients, including pain, fatigue, sleep disturbances, anxiety, depression, perceived stress (PSS), cognitive dysfunction, and fear of recurrence (FOR), greatly affecting their quality of life. The evidence indicates that anxiety and depression are significantly correlated with a decreased quality of life in breast cancer patients [5].

Malignant tumors have a significant psychological impact on patients, and women are often more delicate and sensitive than men. Thus, female patients experience enormous mental pressure either upon learning of their malignant tumors or during the follow-up treatment[6]. Emotions are contagious, and the emotional state of cancer patients profoundly impacts the family around them and their daily activities. Negative and depressed emotions not only cast a shadow on the entire family[7] but also make it difficult for patients to cooperate with doctors with a positive attitude, seriously reducing their quality of life. As the most common malignant tumor with the highest incidence among women[8], ~~the harm of~~ breast cancer to female patients cannot be ignored. Therefore, improving the psychological status of breast cancer patients is of great significance.

As a psychologically focused therapy, mindfulness-based stress reduction (MBSR) can positively affect stress management ~~in the brain through mindfulness meditation.~~ Patients can better manage their emotions, ~~awaken~~ improve their inner focus, ~~and~~ regulate their mental state with MBSR, alleviating negative emotions such as loneliness, anxiety, and depression. MBSR is a widely used mindfulness-based intervention with main components such as body ~~scan~~, awareness, breathing meditation, walking

meditation, mindful movement, and psychoeducation designed to relieve pain or reduce stress[9, 10]. I~~t is an~~ eight-week group-based therapy ~~that~~ teaches mindfulness [An eight week] skills through various practices, including mindfulness of breath, thoughts, bodily sensations, sounds, and everyday activities, with weekly 2.5-hour sessions and one full retreat day. Participants are given a CD containing instructions for home practice for 45 minutes per day, six days a week[11]. A growing body of robust evidence from several studies has demonstrated that MBSR has a significant effect on improving mental health[12, 13] and is beneficial for psychological states[14-19], anxiety[20], stress[21], depression[22], and pain[23, 24], particularly in high-risk pregnant women[13].

Although the latest meta-analysis suggests a slight improvement in the quality of life through MBSR[25], it is based on data up to April 2018, omitting several relevant randomized controlled trials (RCTs)[15, 26-28] and lacking information on fatigue and sleep quality. Thus, our systematic review and meta-analysis aim to comprehensively evaluate the impact of mindfulness-based stress reduction therapy on the quality of life and psychological well-being of breast cancer patients. Utilizing recent RCTs, this study seeks to provide ~~up-to-date~~ [recent] evidence for the clinical application of MBSR.

2. Materials and Methods

This systematic review and meta-analysis followed the criteria recommended by the Cochrane Collaboration and adhered to the Preferred Reporting Items for Systematic Review and Meta-Analysis (PRISMA) guidelines for reporting the

results[29]. Since this study relied on published data, obtaining consent from participants was not applicable. The meta-analysis is registered with the PROSPERO database under registration number CRD42023459075.

2.1 Literature search strategy

The population, intervention, control, and outcomes (PICO) criteria in this study were determined by the coauthors as follows: assessing the quality of life in breast cancer patients treated with MBSR compared to those receiving ~~usual~~ standard care. We conducted an analysis of both short-term and long-term quality of life in breast cancer patients. ~~To~~ In order to identify relevant studies addressing the impact of MBSR in breast cancer patients, we conducted an integrated search of databases, including Web of Science (from 1946 to July 2023), PubMed (from 1966 to July 2023), Embase (from 1974 to July 2023), China's National Knowledge Infrastructure (from 1976 to July 2023), and the Central Cochrane Registry of Controlled Trials (from 1997 to July 2023). The search terms used were combined to capture relevant studies: (("Mindfulness"[Mesh]) OR ((("mindfulness training") OR ("Mindfulness-based Cognitive Therapy")) OR ("mindfulness-based stress reduction"))) AND ((((((breast cancer [Title/Abstract])) OR ("breast tumor")) OR ("mammary tumor")) OR ("mammary neoplasms ")) OR ("breast neoplasms")). The ~~endpoint~~ aim of our study was to evaluate the improvement in the quality of life in breast cancer patients treated with MBSR using a meta-analysis and to compare with those of patients receiving usual care.

2.2 Inclusion and exclusion criteria

We assessed the quality of life using 11 outcome measures for comparison.

Studies were deemed eligible if they included one of the following outcome measures: perceived pressure (PSS), depression, anxiety, fear of relapse (FOR), coping capacity, quality of life (QOL), sleep quality, post-traumatic growth (PTG), fatigue, pain, and emotional state. In the included studies, mindfulness-based stress reduction was implemented in the experimental group, while usual care was provided in the control group. We excluded meeting notes and abstracts lacking complete RCTs, as well as studies with incomplete data. In cases of replicated published studies, we selected articles with available data and the most recent results. To ensure a comprehensive search for data, we also reviewed the references of the included studies.

2.3 Data Extraction

In this study, two researchers independently screened the literature and completed data extraction. The extracted information included the first author's name, year of publication, country, sample size, intervention details, treatment duration, age, key elements of bias risk assessment, and outcome indicator data. The extracted results were then cross-checked. Any disagreements during data extraction were resolved through discussion and adjudicated by a third senior investigator. For literature lacking original data, we attempted to contact the authors to obtain the raw data; otherwise, the studies were excluded.

2.4 Quality and risk of bias assessment

All included studies underwent quality assessment using the JADAD scale, which evaluates four aspects: random sequence generation, random hiding, blinding, and exit. The tool assigns ratings on a scale of 1 to 7, with scores of 1 to 3 considered

low-quality literature and scores of 4 to 7 considered high-quality literature. To assess the risk of bias, we utilized the Cochrane Bias tool for RCTs, which includes seven assessments: sequence generation, assignment hiding, subject blindness, outcome evaluators, exit and loss of follow-up, incomplete outcome data, and selective outcome reporting. The included literature was categorized as low risk, high risk, or unclear. Two independent reviewers conducted the assessment, and any discrepancies were resolved through negotiation with a third researcher.

2.5 Outcomes

A meta-analysis of 11 outcome indicators was performed through literature integration. Primary outcome measures included perceived stress (PSS), depression, anxiety, fear of relapse (FOR), and coping ability. Secondary outcome measures included quality of life, sleep quality, post-traumatic growth (PTG), fatigue, pain, and emotional state.

2.6 Statistical Analysis

Data were analyzed using Revman 5.3 software. Results for continuous data are presented as mean difference (MD) and 95% confidence interval (CI). Heterogeneity among studies was assessed using the χ^2 and I^2 tests. A P value for the Q statistic of < 0.10 and $I^2 > 50\%$ indicated significant heterogeneity, prompting the use of a random-effects model. Subgroup analysis was conducted to explore the effects of publication date, number of participants, and duration of treatment. In cases where the data from 11 articles were expressed as a median, the algorithm of Hozo et al. [31] was employed to estimate the weighted mean and standard deviation. The test for overall

effects determined statistical significance by the magnitude of the p-value, considering data as statistically significant when $p < 0.05$. Publication bias was assessed using funnel plots.

3. Results

3.1 Results of the search

The RCTs screening process for meta-analysis is illustrated in Figure 1. Initially, a systematic search yielded 1599 articles, which was reduced to 1138 after removing duplicates. Subsequently, a title and abstract filter excluded 205 non-RCTs, 16 conference abstracts, 80 meta-analysis papers, 194 irrelevant documents, 1 animal study, and 167 articles involving non-breast cancer patients. An additional 282 papers not using mindfulness-based stress reduction as an intervention were excluded. Screening the full text of the remaining 193 papers led to the exclusion of 152 duplicate references, 28 papers lacking the required outcome indicators, and 2 papers with incomplete data. Finally, 11 papers were included in this study.

3.2 Characteristics of Included Studies

A total of 11 randomized controlled trials met our inclusion criteria, and detailed features of the included literature are presented in Table 1. The papers were published between 2009[30] and 2022[28]. Seven studies were conducted in North America countries[26, 27, 30-34], two in ~~the~~ China[28, 35] and two ~~in~~ European ~~countries~~ studies[36, 37]. A total of 1644 patients were included in our study, among which the study by Lengacher et al. [33] included 322 patients (the highest number of cases), while Zhang et al.[35] included 60 patients (the lowest number of cases). Four articles[30,

32-34] used mindfulness-based stress reduction therapy ~~to intervene~~ for 6 weeks, while the remaining seven articles[26-28, 31, 35-37] for 8 weeks. Five articles[26, 32-

used mindfulness-based stress reduction therapy

34, 36] reported on patients with stage 0-III breast cancer, three [28, 30, 37] reported on patients with breast cancer during chemotherapy, one [27] reported on neuropathic pain in breast cancer patients, and the remaining two articles focused on stage I-II[31] and stage I-III [35] breast cancer patients, respectively.

Table 1. Characteristics of the included studies.

First Author	Country	Participants	Intervention methods		Number of patients (Intervention/control)	Treatment duration	Age Ratio		Follow-up time	JADAD	Outcomes
			Observation group	Control group			Observation group	Control group			
Henderson2012	USA	Stage I-II breast cancer	①For 8 weeks, 7 sessions per week of 2.5 to 3.5 hours of therapy and the 6th session of 7.5 hours of intensive silent retreat therapy.② After completing the MBSR, participants are offered three 2-hour sessions per month.	Usual care	53/58	8 weeks	49.8±8.4	49.8 ± 8.4	24 months	4	Coping Capacity
Hoffman 2012	UK	Patients with stage 0-III breast cancer who have completed treatment for 2 months to 2 years	①Courses lasting 8 weeks, 2 hours per week (the first and last courses are 2.25 hours).②Week 6 plus a 6-hour mindfulness session. Do home exercises for 40 to 45 minutes six or seven days a week.	Usual care	114/115	8 weeks	49.0±9.3	50.1±9.1	3 months	6	Emotional States
Janusek2019	USA	Breast cancer	①For 8 weeks (2.5 hours per week).② 6 hour meditation retreat after the fifth week.	ACC Group: Group education lasting 8 weeks, 2.5 hours per week	63/61	8 weeks	55.0 ± 10.1	55.2 ± 10.1	6 months	7	Sleep Quality、PSS、Depression、Fatigue
Lengacher2009	USA	Stage 0-III	①Two hour sessions six times a week	Routine care,	41/43	6 weeks	56.1±9.1	58.0±1	6 weeks	4	FOR、

		breast cancer patients receiving radiotherapy or chemotherapy after surgery	for six weeks.②Practice meditation techniques for 15-45 minutes a day.	providing mindfulness-based stress reduction after the intervention.				0.2			Anxiety、Depression、PSS、QOL
Lengacher2015	USA	Stage 0 to III breast cancer patients	①Two hour sessions six times a week for six weeks.②Practice meditation techniques for 15-45 minutes a day.	Routine care, providing mindfulness-based stress reduction after the intervention.	38/41	6 weeks	56.1±9.1	58.0±10.2	3 months	5	Sleep Quality
Lengacher2016	USA	Stage 0 to III breast cancer patients	①For 6 weeks, attend a 2-hour course once a week.②Practice 15 to 45 minutes a day.	Routine care, providing mindfulness-based stress reduction after the intervention.	155/167	6 weeks	56.5±10.2	57.6±9.2	3 months	6	Pain、Fatigue、Depression、Anxiety、PSS、FOR
Reich2017	USA	Stage 0 to III breast cancer patients	①A two-week course in formal meditation techniques.②Practice 15-45 minutes a day.	Usual care	167/155	6 weeks	56.6	56.6	3 months	4	Depression、PSS、Sleep Quality、Anxiety

											、QOL、Fatigue、Pain、FOR
Sarenmaim2017	Sweden	Breast cancer patients during chemotherapy	①Self-directed mindfulness-based stress reduction course lasting 8 weeks. ②Teachers and weekly group meetings.	Usual care	62/52	8 weeks	57.2±10.2	57.2±10.2	3 months	7	QOL、PTG、Coping Capacity
Shergill2022	Canada	Breast cancer survivors with nerve pain	①Courses lasting 8 weeks, 2.5 hours per week.②One day (about 6 hours) of retreat a week.	Usual care	49/49	8 weeks	51.3±11.4	55.1±9.6	3 months	7	PSS、Pain、Emotional States
Zhang2017	China	Stage I-III breast cancer patients	①For 8 weeks, 2 hours of treatment per week.②40-45 minutes of homework 6 or 7 days a week.	Usual care	30/30	8 weeks	48.67 ± 8.49	46.00 ± 5.12	3 months	6	PSS、Anxiety、PTG
Zhu2022	China	Breast cancer patients who have completed their first chemotherapy after surgery	Mindfulness training was performed six days a week (at least 30 minutes) for eight weeks.	Usual care	50/51	8 weeks	47.96 ± 8.51	49.78 ± 7.48	/	6	PTG

3.2.1 Participants and Interventions

The details are given in Table 1.

3.2.2 Conflicts of Interest

For all studies included, the authors indicated no potential conflicts of interest.

3.2 Primary Outcomes

3.2.1 Perceived Stress

The perceived stress (PSS) is a 10-item scale that assesses the degree to which life experiences are appraised as uncontrollable[38]. Four studies[26, 30, 34, 35] reported the PSS (Figure 2). A meta-analysis showed that MBSR therapy significantly reduced PSS in breast cancer patients compared to usual care (MD = -1.46，95%CI = -2.53 to -0.38，$p = 0.03$), showed high heterogeneity ($I^2 = 67\%$，$p < 0.00001$).

We performed a subgroup analysis based on publication date and number of participants. Subgroup analysis (Table 2) showed that MBSR had a significant advantage over usual care in reducing PSS in breast cancer patients. Three articles [34, 39, 40] reported stress in patients with breast cancer after 3-6 months of follow-up (Figure 3), after 3-6 months of follow-up, MBSR showed no difference in improving PSS in breast cancer patients compared to usual care (MD = -1.62，95%CI = -3.48 to 0.23，$p = 0.08$), showed high heterogeneity ($I^2 = 60\%$，$p = 0.010$).

Figure 2: The forest plots of primary outcomes. (A) HQOL (B) Depression (C) Anxiety (D) Perceived Pressure (E) Personal Growth (F) Fear of relapse.

Figure 3: The forest plot of primary outcomes after follow-up. (A)HQOL(B) Depression (C) Anxiety (D) Perceived Pressure.

3.2.2 Depression

Depressive symptoms were measured by the 20-item Center for Epidemiological Studies Depression Scale[41]. Three studies[26, 30, 33] reported the depression (Figure 2). A meta-analysis showed that MBSR therapy significantly reduced depression in breast cancer patients compared to usual care (MD = -1.84，

95%CI = -3.99 to -0.30，$p = 0.0004$), showed high heterogeneity ($I^2 = 87\%$，$p < 0.00001$). Subsequently, we performed a subgroup analysis based on publication date and number of participants. Subgroup analysis (Table 3) showed that MBSR was more effective than usual care in reducing depression in breast cancer patients. Two articles [33, 39] reported depression in patients with breast cancer after 3-6 months of follow-up (Figure 3), after 3-6 months of follow-up, MBSR showed no difference in improving depression in breast cancer patients compared to the usual care (MD = -0.26，95%CI = -1.62 to 1.09，$p = 0.93$), showed low heterogeneity ($I^2 = 0\%$，$p = 0.70$).

Table 2. Subgroup analysis of PSS.

Subgroups	PSS						
	Studies, n	Participants, n	I^2	Q-test	Mean difference	95% CI	*P*
Publication date							
＞2017 or = 2017	2	444	56	0.13	-0.75	-2.63,1.12	0.43
＜2017	2	140	0	0.48	-1.82	-2.12,-1.52	＜0.00001
Treatment duration							
＜7 weeks	2	389	88	0.004	-1.69	-1.98,-1.39	＜0.00001
＞7 weeks	2	195	0	0.74	-2.30	-3.78,-0.81	0.002

3.2.3 Anxiety

Anxiety measured by the State-Trait Anxiety Inventory, higher scores are indicative of more anxiety. Three studies[30, 33, 35] reported the anxiety (Figure 2). A meta-analysis showed that MBSR therapy significantly reduced anxiety in breast cancer patients compared to usual care (MD = -2.81，95%CI = -5.31 to -0.32，p = 0.002)， showed high heterogeneity (I^2 = 84%，p < 0.00001). We performed a subgroup analysis on the number of participants. Subgroup analysis (Table 3) showed that MBSR was more effective than ~~usual~~ standard care in reducing anxiety in breast cancer patients. Two articles [33, 40] reported anxiety in patients with breast cancer after 3 months of follow-up (Figure 3), after 3 months of follow-up, MBSR showed no difference in improving anxiety in breast cancer patients compared to usual care (MD = -2.40，95%CI = -3.98 to -0.83，p = 0.32), showed low heterogeneity (I^2= 1%，p = 0.003).

3.2.4 Fear of Relapse

Fear of relapse (FOR) measured by the 30-item Concerns about Recurrence Scale[42]. Two studies[30, 33] reported the FOR (Figure 2). The results of the meta-analysis showed that MBSR therapy ~~was~~ were significantly better than ~~usual~~ standard care in reducing FOR in breast cancer patients (MD = -1.27，95%CI = -3.44 to 0.90，p = 0.0004)， showed high heterogeneity (I^2 = 92%，p < 0.00001).

3.2.5 Coping Capacity

Coping capacity was evaluated using the Sense of Coherence scale (SOC)[43]. Two studies[31, 37] reported the coping capacity (Figure 2). The results of the meta-analysis showed that MBSR therapy was significantly better than usual care in improving the coping capacity in breast cancer patients (MD = 1.26, 95%CI = -3.23 to5.76, p = 0.03), showed high heterogeneity (I^2 = 78%，p < 0.00001).

3.3 The Secondary Outcome

3.3.1 Quality of Life

Quality of Life (QOL) was measured by the Medical Outcomes Studies Short-form General Health Survey[44]. Four studies[30, 33, 34, 37] reported the QOL (Figure 4), and a meta-analysis showed that MBSR therapy did not make a significant difference in improving QOL compared to usual care (MD =

1.81, 95%CI = -0.46 to 4.08, p = 0.22), showed low heterogeneity (I^2= 33%, $p < 0.00001$). Subsequently, we performed a subgroup analysis based on publication date and number of participants. A subgroup analysis (Table 4) showed that MBSR was no less effective than usual care in improving QOL for breast cancer patients. Two articles[33, 34] reported QOL after 3 months of follow-up (Figure 5), after three months of follow-up, MBSR did not differ in improving QOL compared to usual care (MD = -0.30, 95%CI = -4.04 to 3.44, p = 0.46), showed low heterogeneity (I^2= 0%, p = 0.88).

Figure 4: The forest plots of secondary outcomes. (A) Sleep Quality (B) Fatigue (C) Pain (D) Coping Capacity (E) Emotional State.

Table 3. Subgroup analysis of depression and anxiety.

Subgroups	Depression							Anxiety						
	Studies, n	Participants, n	I^2	Q-test	Mean difference	95% CI	*P*	Studies, n	Participants, n	I^2	Q-test	Mean difference	95% CI	*P*
Publication date														
＞2017 or = 2017	2	437	0	0.88	-0.74	-1.95,0.47	0.23							
＜2017	2	382	93	0.0002	-2.08	-4.62,0.47	＜ 0.00001							
Number of participants														
＜100	2	219	50	0.16	-2.7	-4.70,-0.69	0.008	2	140	88	0.004	-1.00	-2.74,0.73	0.26
＞100	2	606	0	1	-0.70	-1.62,0.22	0.14	2	622	0	0.74	-2.30	-3.78,-0.81	0.002

Table 4. Subgroup analysis of QOL.

Subgroups	Studies, n	Participants, n	I^2	Q-test	Mean difference	95% CI	*P*
Publication date							
＞2017 or = 2017	2	411	0	0.97	-0.41	-4.88,4.07	0.86
＜2017	2	379	53	0.14	2.21	2.41,3.81	0.16
Number of participants							
＜100	2	196	0	0.33	3.16	2.45,3.86	＜0.00001
＞100	2	594	0	0.97	-0.40	-4.05,3.24	0.83

3.3.2 Sleep Quality

Sleep quality was measured by the Pittsburgh Sleep Quality Index[45]. Three studies[26, 32, 34] reported the sleep quality (Figure 4). Meta-analysis showed that there was no significant difference between MBSR therapy and usual care in improving the sleep quality of breast cancer patients (MD = -0.43, 95%CI = -0.67 to -0.19, $p = 0.69$)， showed low heterogeneity ($I^2 = 0\%$，$p = 0.0004$). Three articles [32, 34, 39] reported sleep quality in patients with breast cancer after 3-6 months of follow-up (Figure 5), after 3-6 months of follow-up, MBSR showed no difference in improving sleep quality in breast cancer patients compared to usual care (MD = -0.45, 95%CI = -0.68 to -0.22，$p = 0.39$), showed low heterogeneity ($I^2 = 0\%$，$p = 0.0001$).

Figure 5: The forest plots of secondary outcomes after follow-up. (A) Sleep Quality (B) Fatigue.

3.3.3 Post-traumatic Growth

Post-traumatic growth (PTG) was evaluated using the Posttraumatic Growth Inventory (PTGI)[46]. Three studies[28, 35, 37] reported PTG (Figure 4)， and meta-analysis results showed that no significant difference could be observed between MBSR therapy and usual care in PTG in breast cancer patients (MD = 7.59，95%CI = 4.23 to 10.96，$p = 0.18$) and the heterogeneity was low ($I^2 = 41\%$，$p < 0.00001$).

3.3.4 Fatigue

Fatigue was measured using the Fatigue Symptom Inventory[47]. Two studies[26, 33] reported fatigue (Figure 4), and meta-analysis results showed that no significant difference could be observed between MBSR therapy and usual care in reducing fatigue in breast cancer patients (MD = -1.01, 95%CI = -2.79 to 0.76, $p = 0.87$). Showed low heterogeneity ($I^2 = 0\%$，$p = 0.26$). Two articles [33, 39] reported fatigue in patients with breast cancer after 3-6 months of follow-up (Figure 5), after 3-6 months of follow-up, MBSR showed no difference in improving fatigue in breast cancer patients compared to usual care (MD = -1.12, 95%CI = -3.03 to 0.79，$p = 0.83$), showed low heterogeneity ($I^2 = 0\%$，$p = 0.25$).

3.3.5 Pain

Pain was measured by the Brief Pain Inventory, which examines pain intensity and interference[48].

Two studies[27, 33] reported the ache (Figure 4), and meta-analysis results showed that no significant difference could be observed between MBSR therapy and ~~usual~~ care in reducing pain in breast cancer patients (MD = 0.32, 95%CI = -0.93 to 1.58, p = 0.21), showed low heterogeneity (I^2 = 36%， p = 0.70).

3.3.6 Emotional States

Emotional states measured by using the Profile of Mood State (POMS). Two studies[27, 36] reported the emotional states (Figure 4). The results of the meta-analysis showed that MBSR therapy was significantly better than ~~usual~~ care in improving the emotional states in breast cancer patients (MD = -7.73, 95%CI = -27.34 to 11.88, p = 0.0007), showed high heterogeneity (I^2 = 91%， p = 0.11).

3.4 Assessment of Risk of Bias

The purpose of funnel plots is to illustrate whether the included studies exhibit significant publication bias (S1 Fig). One article[36] did not provide a specific description of the double-blind method, so its type was categorized as high-risk. Four articles[30-32, 34] did not describe the method of concealment of allocation, but the remaining articles used characteristics such as hidden opaque envelopes or bottles for allocation. Four papers[30, 31, 34, 35] did not detail the randomization method and were assessed as unclear. Additionally, one article[35] reported incomplete outcome data and was rated as high risk.

4. Discussion

4.1 Main results and connection with other studies

A meta-analysis of 11 outcome measures, including stress, depression, anxiety, coping ability and fear of relapse, quality of life, sleep quality, post-traumatic growth, fatigue, pain, and emotional state, was conducted through literature integration.

The results of this study revealed that MBSR therapy showed no significant difference in both short- and long-term QOL for breast cancer patients compared to usual care. This aligns with the findings of a meta-analysis by Zhang et al. [49]. In the meta-analysis of Haller et al. [50], MBSR therapy demonstrated a short-term positive effect on improving the QOL of breast cancer patients. The divergence in results could be attributed to the inclusion of a broader range of quality of life scoring tools in the Haller et al. study,

while our study used a single assessment tool with eight dimensions. Not every dimension may necessarily be meaningful, contributing to the observed heterogeneity. Sarenmalm et al. [37] discovered that MBSR can enhance the short-term QOL of breast cancer patients. This difference may be attributed to the intervention duration in the study, which was 8 weeks, compared to the 6-week duration of MBSR in Lengacher et al. [30] and Reich et al. [51]. It suggests that only long-term MBSR therapy may have a positive impact on the short-term QOL of breast cancer patients.

An increasing number of doctors and nurses are turning to mindfulness-based stress reduction therapy to alleviate the negative emotions of breast cancer patients. The findings suggest that MBSR therapy effectively improves anxiety, depression, and emotional state in breast cancer patients after intervention, aligning with the findings of Hoffman et al.[52]. This positive impact on emotions is attributed to the core element of MBSR intervention, which involves meditation—a mind-based practice designed to achieve a balanced mental state[53]. ~~This~~ Furthermore, this positive impact on emotions is attributed to the core element of MBSR intervention, which involves meditation—a mind-based practice designed to achieve a balanced mental state[50]. This implies that while MBSR therapy can offer short-term relief for negative emotions in breast cancer patients, its long-term efficacy is less apparent. ~~The~~ A potential explanation is that the initial improvement in the status of breast cancer patients after MBSR intervention diminishes over time, overshadowed by the gradual increase in adverse reactions during treatment. Regarding promoting ~~P~~p ost-traumatic growth in breast cancer patients, MBSR shows no significant difference compared to ~~usual~~ care, but there is a trend toward improvement. The analysis suggests that the limited number of included studies and insufficient sample size may contribute to this observed difference. The results indicate that MBSR does not significantly enhance short- and long-term sleep quality or reduce distress in breast cancer patients compared to ~~usual~~ care, consistent with the meta-analysis results by Zhang et al.[49]. The study of Janusek et al. [26] found that MBSR intervention could improve the sleep quality of breast cancer patients in the short term after 8 weeks, whereas interventions of 6 weeks by Lengacher et al. [54] and Reich et al. [51] showed less significant effects. This suggests that insufficient intervention time might impact the improvement of sleep quality in breast cancer patients. Furthermore, the study results indicate no significant

difference in reducing fatigue and pain in breast cancer patients with MBSR compared to usual care, potentially influenced by different disease stages. During chemotherapy, breast cancer patients may experience aggravated tumor-related symptoms, new symptoms, and an increased likelihood of psychiatric symptoms. On a positive note, the study demonstrates that MBSR significantly enhances coping skills in breast cancer patients, measured through the Sense of Coherence (SOC). ~~SOC~~ reflects an individual's general perception and feeling of the outside world, representing a stable and lasting self-confidence. Higher SOC correlates with an improved ability to utilize resources and cope with pressure. Patients with higher SOC reported fewer mental symptoms, such as anxiety and depression, with a significant reduction in the prevalence of depression. The analysis attributes this improvement to MBSR techniques, such as meditation and yoga, which enhance concentration and coping skills, redirecting patients toward a healthier self-awareness and self-relationship.

MBSR demonstrated a significant reduction in negative emotions, including anxiety, depression, stress, and fear of relapse in breast cancer patients. However, there were no significant differences compared to usual care in terms of improving quality of life, sleep quality, post-traumatic growth, pain, and fatigue. All outcome indicators in this study were measured using a uniform tool, contributing to a limited number of included studies. Moving forward, it is essential to conduct large-sample randomized controlled trials, establishing a standardized evaluation method for outcome indicators. This will contribute to evidence-based insights and a clearer understanding of the impact of MBSR on breast cancer patients.

4.2 Clinical Implications

~~As an adjunctive treatment, mindfulness-based stress reduction (MBSR) proves effective in significantly reducing~~ Previous studies point to MBSR significantly reducing negative emotions such as anxiety, depression, stress, and fear in breast cancer patients. ~~It~~ Also, MBSR enhances patients' coping abilities and positively influences their emotional states. Moreover, MBSR is characterized by its low threshold for use, simplicity in implementation, and low economic cost, making it suitable for post-disease psychological intervention. ~~Its application~~ Applying MBSR can contribute to a more comprehensive and improved treatment system for breast cancer patients.

4.3 Limitations

There are limitations to our paper. The main limitation was the heterogeneity of patient populations in terms of inclusion criteria and clinical features. Breast cancer patients may be at different stages of their illness, which could be an important confounder. Different conditions and prior therapies could also influence the results. For some patients in the early stage of the disease, surgical treatment can resolve the disease, while patients in the advanced stage of the disease may be followed by targeted therapy or immunotherapy. No study was precise enough to assess whether treatment measures affect the effectiveness of MBSR at different stages of treatment. In addition, the number of outcome indicators is inconsistent between different studies, and some studies lack sufficient indicators. This variability could lead to incomplete data interpretation. As there was measurable significant heterogeneity across the studies, our conclusions should still be interpreted with caution.

5. Conclusion

In conclusion, ~~Mindfulness-Based Stress Reduction (~~MBSR~~)~~ emerges as an effective intervention in significantly reducing negative emotions, including anxiety, depression, stress, and fear of relapse in breast cancer patients. ~~It also~~ Our study also demonstrates positive effects on enhancing patients' coping skills and emotional well-being. However, to strengthen the evidence base, further trials with extended follow-up periods and more tightly controlled conditions are warranted.

Acknowledgments

Not applicable.

Author Contributions

Drafted the article, X.W. (Xiaohui Wang) and X.Z. (Xinying Zhu); methodology, X.W. and Z.D. (Zhicheng Dai) and Y.L. (Yu Li) and T.Z. (Tongxia Zhan); data curation, X.W., Y.L., X.C. (Xinghui Cui) and L.M. (Limin Ma); writing—review and editing, T.Z., X.C. and L.M.; All authors have read and agreed to the published version of the manuscript.

Institutional Review Board Statement

Not applicable.

Informed Consent Statement

Not applicable.

Data Availability Statement

Data will be provided upon request.

Conflicts of Interest

The authors declare no conflict of interest.

Funding

This work was supported by the Weifang Science and Technology Development Plan college class [grant number 2021GX061]、Research Fund Project of School of Nursing, Weifang Medical University [grant number 2022MS004] and Weifang Medical College Nursing Research Fund 2022 annual project, 2022MS003

Figure legends

Figure 1: Flow chart of study selection.

Figure 2: The forest plots of primary outcomes. (A) HQOL (B) Depression (C) Anxiety (D) Perceived Pressure (E) Personal Growth (F) Fear of relapse.

Figure 3: The forest plot of primary outcomes after follow-up. (A)HQOL(B) Depression (C) Anxiety (D) Perceived Pressure.

Figure 4: The forest plots of secondary outcomes. (A) Sleep Quality (B) Fatigue (C) Pain (D) Coping Capacity (E) Emotional State.

Figure 5: The forest plots of secondary outcomes after follow-up. (A) Sleep Quality (B) Fatigue.

Table legends

Table 1: Characteristics of the included studies.

Table 2: Subgroup analysis of HQOL and Depression.

Table 3: Subgroup analysis of Anxiety.

Table 4: Subgroup analysis of PSS.

References

1. Soriano-Maldonado A, Díez-Fernández DM, Esteban-Simón A, Rodríguez-Pérez MA, Artés-Rodríguez E, Casimiro-Artés MA, et al. Effects of a 12-week supervised resistance training program, combined with home-based physical activity, on physical fitness and quality of life in female breast cancer survivors: the EFICAN randomized controlled trial. J Cancer Surviv. 2023;17(5):1371-85. Epub 2022/03/23. doi: 10.1007/s11764-022-01192-1. PubMed PMID: 35314958; PubMed Central PMCID: PMCPMC10442259.
2. Harbeck N, Gnant M. Breast cancer. Lancet. 2017;389(10074):1134-50. Epub 2016/11/21. doi: 10.1016/s0140-6736(16)31891-8. PubMed PMID: 27865536.
3. Christensen RAG, Haykowsky MJ, Nadler M, Prado CM, Small SD, Rickard JN, et al. Rationale and design of IMPACT-women: a randomised controlled trial of the effect of time-restricted eating, healthy eating and reduced sedentary behaviour on metabolic health during chemotherapy for early-stage breast cancer. Br J Nutr. 2023;130(5):852-9. Epub 2022/12/02. doi: 10.1017/s0007114522003816. PubMed PMID: 36453589; PubMed Central PMCID: PMCPMC10404477.
4. Yusof KM, Mohd Sidik S, Mahmud R, Abdullah M, Avery-Kiejda KA, Rosli R. Association of psychological distress with arm morbidity symptoms in breast cancer survivors: outcomes from the use of PHQ-9 and GAD-7 questionnaires. Breast Cancer. 2023;30(5):810-9. Epub 2023/06/12. doi: 10.1007/s12282-023-01475-0. PubMed PMID: 37306933.
5. Liu W, Liu J, Ma L, Chen J. Effect of mindfulness yoga on anxiety and depression in early breast cancer patients received adjuvant chemotherapy: a randomized clinical trial. J Cancer Res Clin Oncol. 2022;148(9):2549-60. Epub 2022/07/06. doi: 10.1007/s00432-022-04167-y. PubMed PMID: 35788727; PubMed Central PMCID: PMCPMC9253261.
6. Mahalq S, Lahlou L, Rammouz I, Abouqal R, Belayachi J. Factors associated with psychological burden of breast cancer in women in Morocco: cross□sectional study. BMC Womens Health. 2023;23(1):590. Epub 20231110. doi: 10.1186/s12905-023-02769-3. PubMed PMID: 37950247; PubMed Central PMCID: PMCPMC10638816.
7. Ammar-Shehada W, Abusaman K, Bracke P. Perceived support, social and marital challenges in the lives of breast cancer survivors after illness: a self-administered cross-sectional survey. Front Sociol. 2023;8:1227529. Epub 20230907. doi: 10.3389/fsoc.2023.1227529. PubMed PMID: 37744401; PubMed Central PMCID: PMCPMC10513897.
8. Sung H, Ferlay J, Siegel RL, Laversanne M, Soerjomataram I, Jemal A, et al. Global Cancer Statistics 2020: GLOBOCAN Estimates of Incidence and Mortality Worldwide for 36 Cancers in 185 Countries. CA Cancer J Clin. 2021;71(3):209-49. Epub 20210204. doi: 10.3322/caac.21660. PubMed PMID: 33538338.
9. Li H, Chen H, Ye Z, Li X, Yang L, Wang S, et al. Effect of the Online Mindfulness-Based Stress Reduction on Anxiety and Depression Status of COVID-19 Patients Treated in Fangcang Hospitals: A Randomized Controlled Trial. Psychol Res Behav Manag. 2023;16:2735-45. Epub 2023/07/24. doi: 10.2147/prbm.S414553. PubMed PMID: 37485280; PubMed Central PMCID: PMCPMC10362863.
10. Kabat-Zinn J. An outpatient program in behavioral medicine for chronic pain patients based on the practice of mindfulness meditation: theoretical considerations and preliminary results. Gen Hosp Psychiatry. 1982;4(1):33-47. Epub 1982/04/01. doi: 10.1016/0163-8343(82)90026-3. PubMed PMID: 7042457.
11. Zainal NZ, Booth S, Huppert FA. The efficacy of mindfulness-based stress reduction on mental health of breast cancer patients: a meta-analysis. Psychooncology. 2013;22(7):1457-65. Epub 2012/09/11. doi: 10.1002/pon.3171. PubMed PMID: 22961994.
12. Grossman P, Niemann L, Schmidt S, Walach H. Mindfulness-based stress reduction and health benefits. A meta-analysis. J Psychosom Res. 2004;57(1):35-43. Epub 2004/07/17. doi: 10.1016/s0022-3999(03)00573-7. PubMed PMID: 15256293.
13. Goetz M, Schiele C, Müller M, Matthies LM, Deutsch TM, Spano C, et al. Effects of a Brief Electronic Mindfulness-Based Intervention on Relieving Prenatal Depression and Anxiety in Hospitalized High-Risk

Pregnant Women: Exploratory Pilot Study. J Med Internet Res. 2020;22(8):e17593. Epub 2020/08/12. doi: 10.2196/17593. PubMed PMID: 32780023; PubMed Central PMCID: PMCPMC7448174.
14. Lerman R, Jarski R, Rea H, Gellish R, Vicini F. Improving symptoms and quality of life of female cancer survivors: a randomized controlled study. Ann Surg Oncol. 2012;19(2):373-8. Epub 2011/09/14. doi: 10.1245/s10434-011-2051-2. PubMed PMID: 21913014.
15. Zhu P, Liu X, Shang X, Chen Y, Chen C, Wu Q. Mindfulness-Based Stress Reduction for Quality of Life, Psychological Distress, and Cognitive Emotion Regulation Strategies in Patients With Breast Cancer Under Early Chemotherapy-a Randomized Controlled Trial. Holist Nurs Pract. 2023;37(3):131-42. doi: 10.1097/HNP.0000000000000580. PubMed PMID: 37070838.
16. Yildirim D, Çiriş Yildiz C, Ozdemir FA, Harman Özdoğan M, Can G. Effects of a Mindfulness-Based Stress Reduction Program on Stress, Depression, and Psychological Well-being in Patients With Cancer: A Single-Blinded Randomized Controlled Trial. Clin Neuropharmacol. 2022. Epub 2022/12/09. doi: 10.1097/ncc.0000000000001173. PubMed PMID: 36480346.
17. Sun L, Zhang X, Gong P, Zhang L, Zhao Y. Clinical Efficacy of Bevacizumab Plus XELOX Chemotherapy in Colorectal Cancer and Application Value of Mindfulness-Based Stress Reduction Intervention. Altern Ther Health Med. 2022;28(6):65-71. Epub 2022/04/23. PubMed PMID: 35452419.
18. Tian X, Yi LJ, Liang CS, Gu L, Peng C, Chen GH, et al. The Impact of Mindfulness-Based Stress Reduction (MBSR) on Psychological Outcomes and Quality of Life in Patients With Lung Cancer: A Meta-Analysis. Front Psychol. 2022;13:901247. Epub 2022/07/16. doi: 10.3389/fpsyg.2022.901247. PubMed PMID: 35837627; PubMed Central PMCID: PMCPMC9274275.
19. Chayadi E, Baes N, Kiropoulos L. The effects of mindfulness-based interventions on symptoms of depression, anxiety, and cancer-related fatigue in oncology patients: A systematic review and meta-analysis. PLoS One. 2022;17(7):e0269519. Epub 20220714. doi: 10.1371/journal.pone.0269519. PubMed PMID: 35834503; PubMed Central PMCID: PMCPMC9282451.
20. Hofmann SG, Sawyer AT, Witt AA, Oh D. The effect of mindfulness-based therapy on anxiety and depression: A meta-analytic review. J Consult Clin Psychol. 2010;78(2):169-83. doi: 10.1037/a0018555. PubMed PMID: 20350028; PubMed Central PMCID: PMCPMC2848393.
21. Chiesa A, Serretti A. Mindfulness-based stress reduction for stress management in healthy people: a review and meta-analysis. J Altern Complement Med. 2009;15(5):593-600. doi: 10.1089/acm.2008.0495. PubMed PMID: 19432513.
22. Kim J, Shin C, Han KM, Lee MS, Jeong HG, Pae CU, et al. Investigating the effectiveness of a smart mental health intervention (inMind) for stress reduction during pharmacological treatment for mild to moderate major depressive disorders: Study protocol for a randomized control trial. Front Psychiatry. 2023;14:1034246. Epub 2023/04/01. doi: 10.3389/fpsyt.2023.1034246. PubMed PMID: 36998625; PubMed Central PMCID: PMCPMC10043187.
23. Estave PM, Margol C, Beeghly S, Anderson R, Shakir M, Coffield A, et al. Mechanisms of mindfulness in patients with migraine: Results of a qualitative study. Headache. 2023;63(3):390-409. Epub 2023/03/01. doi: 10.1111/head.14481. PubMed PMID: 36853655; PubMed Central PMCID: PMCPMC10088163.
24. Wielgosz J, Kral TRA, Perlman DM, Mumford JA, Wager TD, Lutz A, et al. Neural Signatures of Pain Modulation in Short-Term and Long-Term Mindfulness Training: A Randomized Active-Control Trial. Am J Psychiatry. 2022;179(10):758-67. Epub 2022/07/29. doi: 10.1176/appi.ajp.21020145. PubMed PMID: 35899379; PubMed Central PMCID: PMCPMC9529779.
25. Schell LK, Monsef I, Wockel A, Skoetz N. Mindfulness-based stress reduction for women diagnosed with breast cancer. Cochrane Database Syst Rev. 2019;3(3):CD011518. Epub 20190327. doi: 10.1002/14651858.CD011518.pub2. PubMed PMID: 30916356; PubMed Central PMCID: PMCPMC6436161.
26. Witek Janusek L, Tell D, Mathews HL. Mindfulness based stress reduction provides psychological benefit and restores immune function of women newly diagnosed with breast cancer: A randomized trial with active control. Brain Behav Immun. 2019;80:358-73. Epub 20190403. doi: 10.1016/j.bbi.2019.04.012. PubMed PMID: 30953776.
27. Shergill Y, Rice DB, Khoo EL, Jarvis V, Zhang T, Taljaard M, et al. Mindfulness-Based Stress Reduction in Breast Cancer Survivors with Chronic Neuropathic Pain: A Randomized Controlled Trial. Pain Res Manag. 2022;2022:4020550. Epub 20220707. doi: 10.1155/2022/4020550. PubMed PMID: 35845983;

PubMed Central PMCID: PMCPMC9282981.
28. Zhu P, Wu Q, Liu X, Chen C, Ji Q, Shang X. Effects of Mindfulness-Based Stress Reduction Therapy on Posttraumatic Growth and Quality of Life in Patients With Breast Cancer Under Early Chemotherapy: A Randomized Controlled Trial. Holist Nurs Pract. 2022. Epub 20220828. doi: 10.1097/HNP.0000000000000538. PubMed PMID: 36001131.
29. Liberati A, Altman DG, Tetzlaff J, Mulrow C, Gøtzsche PC, Ioannidis JP, et al. The PRISMA statement for reporting systematic reviews and meta-analyses of studies that evaluate healthcare interventions: explanation and elaboration. Bmj. 2009;339:b2700. Epub 2009/07/23. doi: 10.1136/bmj.b2700. PubMed PMID: 19622552; PubMed Central PMCID: PMCPMC2714672.
30. Lengacher CA, Johnson-Mallard V, Post-White J, Moscoso MS, Jacobsen PB, Klein TW, et al. Randomized controlled trial of mindfulness-based stress reduction (MBSR) for survivors of breast cancer. Psychooncology. 2009;18(12):1261-72. Epub 2009/02/24. doi: 10.1002/pon.1529. PubMed PMID: 19235193.
31. Henderson V, Clemow L, Massion A, Hurley T, Druker S, Hebert JR. The effects of mindfulness-based stress reduction on psychosocial outcomes and quality of life in early-stage breast cancer patients: a randomized trial. Breast Cancer Research and Treatment. 2012;131(1):99-109. doi: 10.1007/s10549-011-1738-1. PubMed PMID: WOS:000298006300010.
32. Lengacher CA, Reich RR, Paterson CL, Jim HS, Ramesar S, Alinat CB, et al. The effects of mindfulness-based stress reduction on objective and subjective sleep parameters in women with breast cancer: a randomized controlled trial. Psycho-Oncology. 2015;24(4):424-32. doi: 10.1002/pon.3603. PubMed PMID: WOS:000352653300008.
33. Lengacher CA, Reich RR, Paterson CL, Ramesar S, Park JY, Alinat C, et al. Examination of Broad Symptom Improvement Resulting From Mindfulness-Based Stress Reduction in Breast Cancer Survivors: A Randomized Controlled Trial. J Clin Oncol. 2016;34(24):2827-34. Epub 2016/06/02. doi: 10.1200/jco.2015.65.7874. PubMed PMID: 27247219; PubMed Central PMCID: PMCPMC5012660 online at www.jco.org. Author contributions are found at the end of this article.
34. Reich RR, Lengacher CA, Alinat CB, Kip KE, Paterson C, Ramesar S, et al. Mindfulness-Based Stress Reduction in Post-treatment Breast Cancer Patients: immediate and Sustained Effects Across Multiple Symptom Clusters.
35. Zhang JY, Zhou YQ, Feng ZW, Fan YN, Zeng GC, Li W. Randomized controlled trial of mindfulness-based stress reduction (MBSR) on posttraumatic growth of Chinese breast cancer survivors. Psychology Health & Medicine. 2017;22(1):94-109. doi: 10.1080/13548506.2016.1146405. PubMed PMID: WOS:000387997900011.
36. Hoffman CJ, Ersser SJ, Hopkinson JB, Nicholls PG, Harrington JE, Thomas PW. Effectiveness of Mindfulness-Based Stress Reduction in Mood, Breast- and Endocrine-Related Quality of Life, and Well-Being in Stage 0 to III Breast Cancer: A Randomized, Controlled Trial. Journal of Clinical Oncology. 2012;30(12):1335-42. doi: 10.1200/jco.2010.34.0331. PubMed PMID: WOS:000303756500019.
37. Kenne Sarenmalm E, Mårtensson LB, Andersson BA, Karlsson P, Bergh I. Mindfulness and its efficacy for psychological and biological responses in women with breast cancer. Cancer Med. 2017;6(5):1108-22. Epub 20170418. doi: 10.1002/cam4.1052. PubMed PMID: 28421677; PubMed Central PMCID: PMCPMC5430085.
38. Cohen S, Kamarck T, Mermelstein R. A Global Measure of Perceived Stress. Journal of Health and Social Behavior. 1983;24(4):385-96. doi: 10.2307/2136404.
39. Janusek LW, Tell D, Mathews HL. Mindfulness based stress reduction provides psychological benefit and restores immune function of women newly diagnosed with breast cancer: A randomized trial with active control. Brain Behavior and Immunity. 2019;80:358-73. doi: 10.1016/j.bbi.2019.04.012. PubMed PMID: WOS:000478105500036.
40. Zhang JY, Zhou YQ, Feng ZW, Fan YN, Zeng GC, Wei L. Randomized controlled trial of mindfulness-based stress reduction (MBSR) on posttraumatic growth of Chinese breast cancer survivors.
41. Radloff LS. The CES-D Scale:A Self-Report Depression Scale for Research in the General Population. Applied Psychological Measurement. 1977;1(3):385-401. doi: 10.1177/014662167700100306.
42. Vickberg SM. The Concerns About Recurrence Scale (CARS): a systematic measure of women's fears about the possibility of breast cancer recurrence. Ann Behav Med. 2003;25(1):16-24. Epub 2003/02/13. doi:

10.1207/s15324796abm2501_03. PubMed PMID: 12581932.
43.Antonovsky A. The structure and properties of the sense of coherence scale. Soc Sci Med. 1993;36(6):725-33. Epub 1993/03/01. doi: 10.1016/0277-9536(93)90033-z. PubMed PMID: 8480217.
44.McHorney CA, Ware JE, Jr., Lu JF, Sherbourne CD. The MOS 36-item Short-Form Health Survey (SF-36): III. Tests of data quality, scaling assumptions, and reliability across diverse patient groups. Med Care. 1994;32(1):40-66. Epub 1994/01/01. doi: 10.1097/00005650-199401000-00004. PubMed PMID: 8277801.
45.Carpenter JS, Andrykowski MA. Psychometric evaluation of the Pittsburgh Sleep Quality Index. J Psychosom Res. 1998;45(1):5-13. Epub 1998/08/28. doi: 10.1016/s0022-3999(97)00298-5. PubMed PMID: 9720850.
46.Tedeschi RG, Calhoun LG. The Posttraumatic Growth Inventory: measuring the positive legacy of trauma. J Trauma Stress. 1996;9(3):455-71. Epub 1996/07/01. doi: 10.1007/bf02103658. PubMed PMID: 8827649.
47.Hann DM, Denniston MM, Baker F. Measurement of fatigue in cancer patients: further validation of the Fatigue Symptom Inventory. Qual Life Res. 2000;9(7):847-54. Epub 2001/04/12. doi: 10.1023/a:1008900413113. PubMed PMID: 11297027.
48.Keller S, Bann CM, Dodd SL, Schein J, Mendoza TR, Cleeland CS. Validity of the brief pain inventory for use in documenting the outcomes of patients with noncancer pain. Clin J Pain. 2004;20(5):309-18. Epub 2004/08/24. doi: 10.1097/00002508-200409000-00005. PubMed PMID: 15322437.
49.Zhang Q, Zhao H, Zheng Y. Effectiveness of mindfulness-based stress reduction (MBSR) on symptom variables and health-related quality of life in breast cancer patients-a systematic review and meta-analysis. Support Care Cancer. 2019;27(3):771-81. Epub 2018/11/30. doi: 10.1007/s00520-018-4570-x. PubMed PMID: 30488223.
50.Haller H, Winkler MM, Klose P, Dobos G, Kümmel S, Cramer H. Mindfulness-based interventions for women with breast cancer: an updated systematic review and meta-analysis. Acta Oncol. 2017;56(12):1665-76. Epub 2017/07/08. doi: 10.1080/0284186x.2017.1342862. PubMed PMID: 28686520.
51.Reich RR, Lengacher CA, Alinat CB, Kip KE, Paterson C, Ramesar S, et al. Mindfulness-Based Stress Reduction in Post-treatment Breast Cancer Patients: Immediate and Sustained Effects Across Multiple Symptom Clusters. J Pain Symptom Manage. 2017;53(1):85-95. Epub 2016/10/11. doi: 10.1016/j.jpainsymman.2016.08.005. PubMed PMID: 27720794; PubMed Central PMCID: PMCPMC7771358.
52.Hoffman CJ, Ersser SJ, Hopkinson JB, Nicholls PG, Harrington JE, Thomas PW. Effectiveness of mindfulness-based stress reduction in mood, breast- and endocrine-related quality of life, and well-being in stage 0 to III breast cancer: a randomized, controlled trial. J Clin Oncol. 2012;30(12):1335-42. Epub 2012/03/21. doi: 10.1200/jco.2010.34.0331. PubMed PMID: 22430268.
53.Deshpande S, Patel KD, Parulkar T, Mahabalesh K, Madhusudhan P, Madhusudhan DK, et al. Effect of Heartfulness meditation based integrative therapy on infertility outcomes: A retrospective case series evaluation. J Ayurveda Integr Med. 2023;14(6):100793. Epub 2023/10/05. doi: 10.1016/j.jaim.2023.100793. PubMed PMID: 37797350; PubMed Central PMCID: PMCPMC10562913.
54.Lengacher CA, Reich RR, Paterson CL, Jim HS, Ramesar S, Alinat CB, et al. The effects of mindfulness-based stress reduction on objective and subjective sleep parameters in women with breast cancer: a randomized controlled trial. Psychooncology. 2015;24(4):424-32. Epub 2014/06/20. doi: 10.1002/pon.3603. PubMed PMID: 24943918; PubMed Central PMCID: PMCPMC4487655.

Supporting Information

S1 Fig：RCT risk of bias summary for included Randomized Controlled Trial.

S2 Fig：The funnel plots of primary outcomes. (A) HQOL(B) Depression (C) Anxiety (D) Perceived

Pressure (E) Personal Growth (F) Fear of relapse.

S3 Fig: The funnel plot of primary outcomes after follow-up. (A)HQOL(B) Depression (C) Anxiety (D) Perceived Pressure.

S4 Fig: The funnel plots of secondary outcomes. (A) Sleep Quality (B) Fatigue (C) Pain (D) Coping Capacity (E) Emotional State.

S5 Fig: The funnel plots of secondary outcomes after follow-up. (A) Sleep Quality (B) Fatigue.

Click here to access/download;Figure;Figure 2.tif

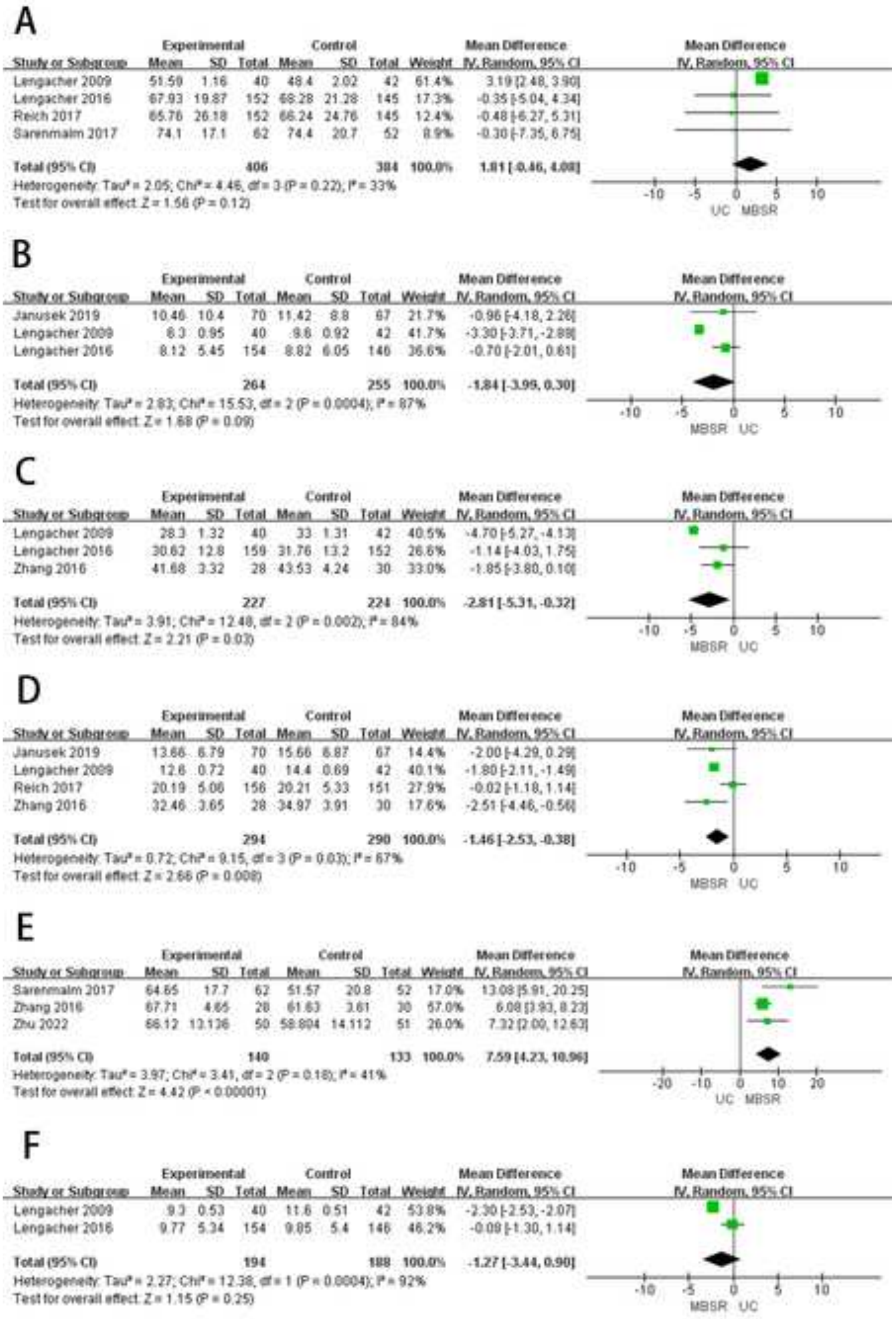

Click here to access/download;Figure;Figure 3.tif

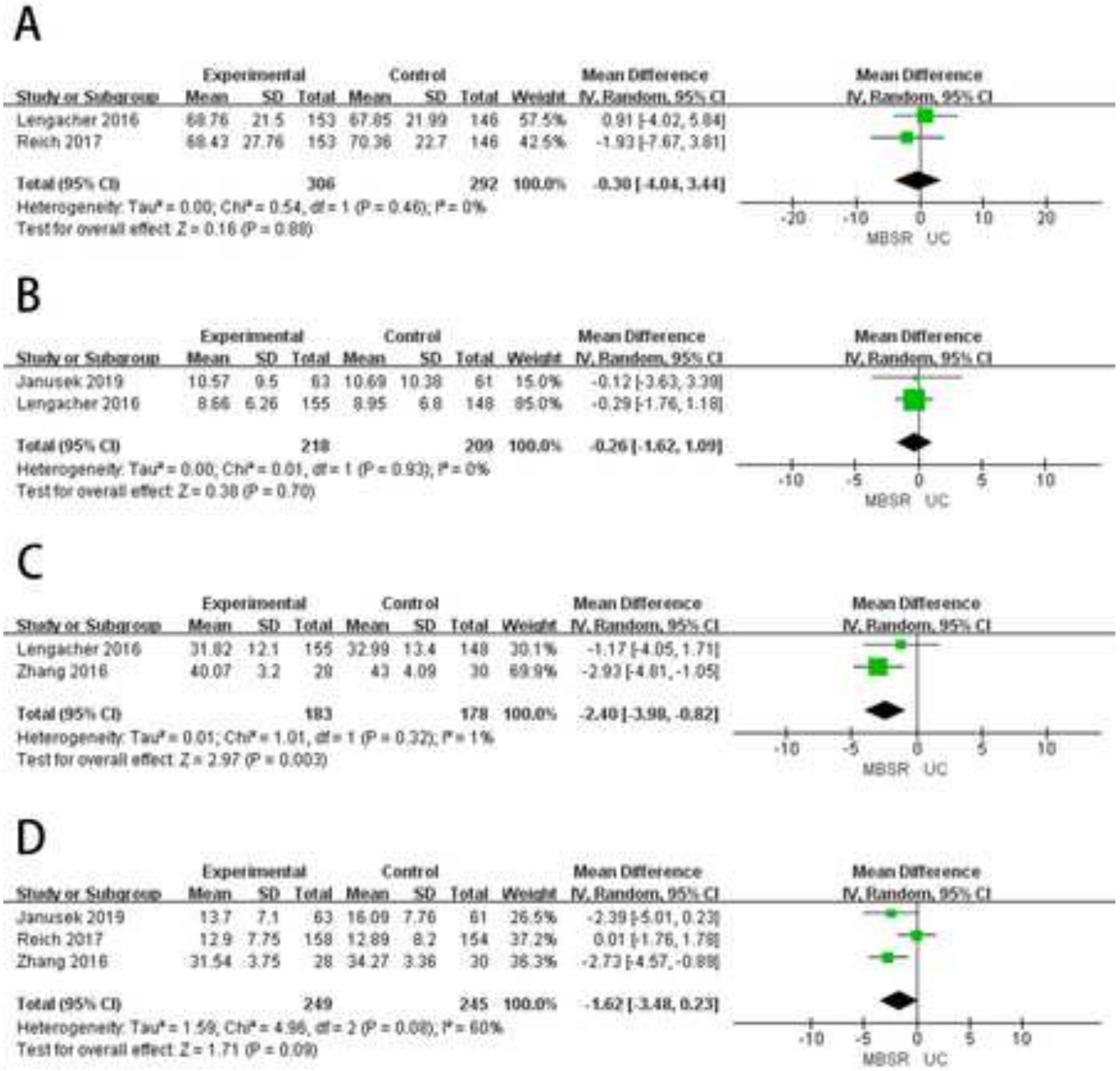

Click here to access/download;Figure;Figure 4.tif

A

Study or Subgroup	Experimental Mean	Experimental SD	Experimental Total	Control Mean	Control SD	Control Total	Weight	Mean Difference IV, Random, 95% CI
Janusek 2019	6.48	3.12	70	7.36	3.55	67	4.5%	-0.88 [-2.00, 0.24]
Lengacher 2015	7.37	0.59	38	7.79	0.55	41	89.6%	-0.42 [-0.67, -0.17]
Reich 2017	7.26	4.47	148	7.52	4.11	145	5.9%	-0.26 [-1.24, 0.72]
**Total (95% CI)**			**256**			**253**	**100.0%**	**-0.43 [-0.67, -0.19]**

Heterogeneity: Tau^2 = 0.00; Chi^2 = 0.74, df = 2 (P = 0.69); I^2 = 0%
Test for overall effect: Z = 3.54 (P = 0.0004)

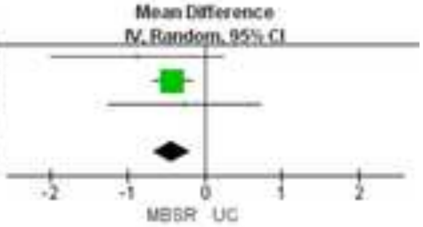

B

Study or Subgroup	Experimental Mean	Experimental SD	Experimental Total	Control Mean	Control SD	Control Total	Weight	Mean Difference IV, Random, 95% CI
Janusek 2019	9.65	23.54	70	10.09	19.98	67	5.9%	-0.44 [-7.74, 6.86]
Lengacher 2016	12.33	7.59	152	13.38	8.45	145	94.1%	-1.05 [-2.88, 0.78]
**Total (95% CI)**			**222**			**212**	**100.0%**	**-1.01 [-2.79, 0.76]**

Heterogeneity: Tau^2 = 0.00; Chi^2 = 0.03, df = 1 (P = 0.87); I^2 = 0%
Test for overall effect: Z = 1.12 (P = 0.26)

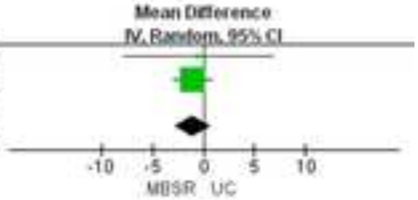

C

Study or Subgroup	Experimental Mean	Experimental SD	Experimental Total	Control Mean	Control SD	Control Total	Weight	Mean Difference IV, Random, 95% CI
Lengacher 2016	9.59	9.44	152	8.28	8.16	151	29.6%	1.31 [-0.68, 3.30]
Shergill 2022	3.49	2.09	49	3.58	2.61	49	70.4%	-0.09 [-1.03, 0.85]
**Total (95% CI)**			**201**			**200**	**100.0%**	**0.32 [-0.93, 1.58]**

Heterogeneity: Tau^2 = 0.35; Chi^2 = 1.56, df = 1 (P = 0.21); I^2 = 36%
Test for overall effect: Z = 0.51 (P = 0.61)

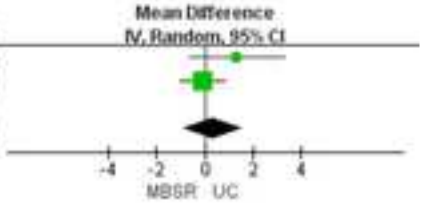

D

Study or Subgroup	Experimental Mean	Experimental SD	Experimental Total	Control Mean	Control SD	Control Total	Weight	Mean Difference IV, Random, 95% CI
Henderson 2012	46.8	1	53	43.7	1	58	60.9%	3.10 [2.73, 3.47]
Sarenmalm 2017	67.7	12	62	69.3	11.5	52	39.1%	-1.60 [-5.92, 2.72]
**Total (95% CI)**			**115**			**110**	**100.0%**	**1.26 [-3.23, 5.76]**

Heterogeneity: Tau^2 = 8.59; Chi^2 = 4.51, df = 1 (P = 0.03); I^2 = 78%
Test for overall effect: Z = 0.55 (P = 0.58)

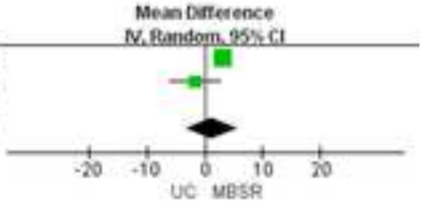

E

Study or Subgroup	Experimental Mean	Experimental SD	Experimental Total	Control Mean	Control SD	Control Total	Weight	Mean Difference IV, Random, 95% CI
Hoffman 2012	30.02	31.6	103	48.08	39.89	111	48.4%	-18.06 [-27.67, -8.45]
Shergill 2022	62.88	16.67	49	60.92	16.46	49	51.6%	1.96 [-4.60, 8.52]
**Total (95% CI)**			**152**			**160**	**100.0%**	**-7.73 [-27.34, 11.88]**

Heterogeneity: Tau^2 = 182.78; Chi^2 = 11.38, df = 1 (P = 0.0007); I^2 = 91%
Test for overall effect: Z = 0.77 (P = 0.44)

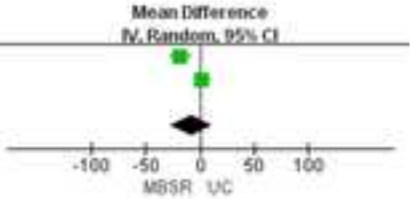

Click here to access/download;Figure;Figure 5.tif

A

Study or Subgroup	Experimental Mean	Experimental SD	Experimental Total	Control Mean	Control SD	Control Total	Weight	Mean Difference IV, Random, 95% CI
Janusek 2019	6.94	3.26	63	6.88	3.58	61	3.7%	0.06 [-1.15, 1.27]
Lengacher 2015	6.91	0.56	38	7.41	0.54	41	90.7%	-0.50 [-0.74, -0.26]
Reich 2017	7.08	4.42	150	7.02	4.12	145	5.6%	0.06 [-0.91, 1.03]
**Total (95% CI)**			**251**			**247**	**100.0%**	**-0.45 [-0.68, -0.22]**

Heterogeneity: $Tau^2 = 0.00$; $Chi^2 = 1.90$, $df = 2$ ($P = 0.39$); $I^2 = 0\%$

Test for overall effect: $Z = 3.79$ ($P = 0.0001$)

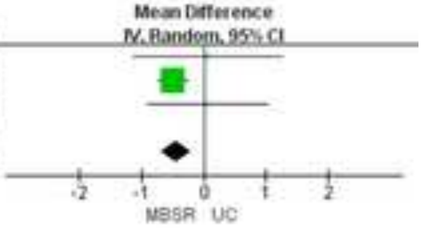

B

Study or Subgroup	Experimental Mean	Experimental SD	Experimental Total	Control Mean	Control SD	Control Total	Weight	Mean Difference IV, Random, 95% CI
Janusek 2019	8.28	22.46	63	10.33	25.58	61	5.1%	-2.05 [-10.53, 6.43]
Lengacher 2016	12.2	8.56	152	13.27	8.71	147	94.9%	-1.07 [-3.03, 0.89]
**Total (95% CI)**			**215**			**208**	**100.0%**	**-1.12 [-3.03, 0.79]**

Heterogeneity: $Tau^2 = 0.00$; $Chi^2 = 0.05$, $df = 1$ ($P = 0.83$); $I^2 = 0\%$

Test for overall effect: $Z = 1.15$ ($P = 0.25$)

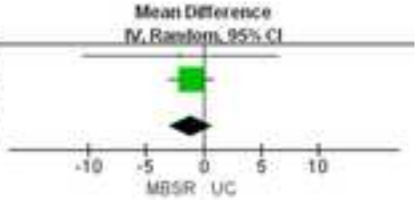

Click here to access/download;Figure;Figure1.tif

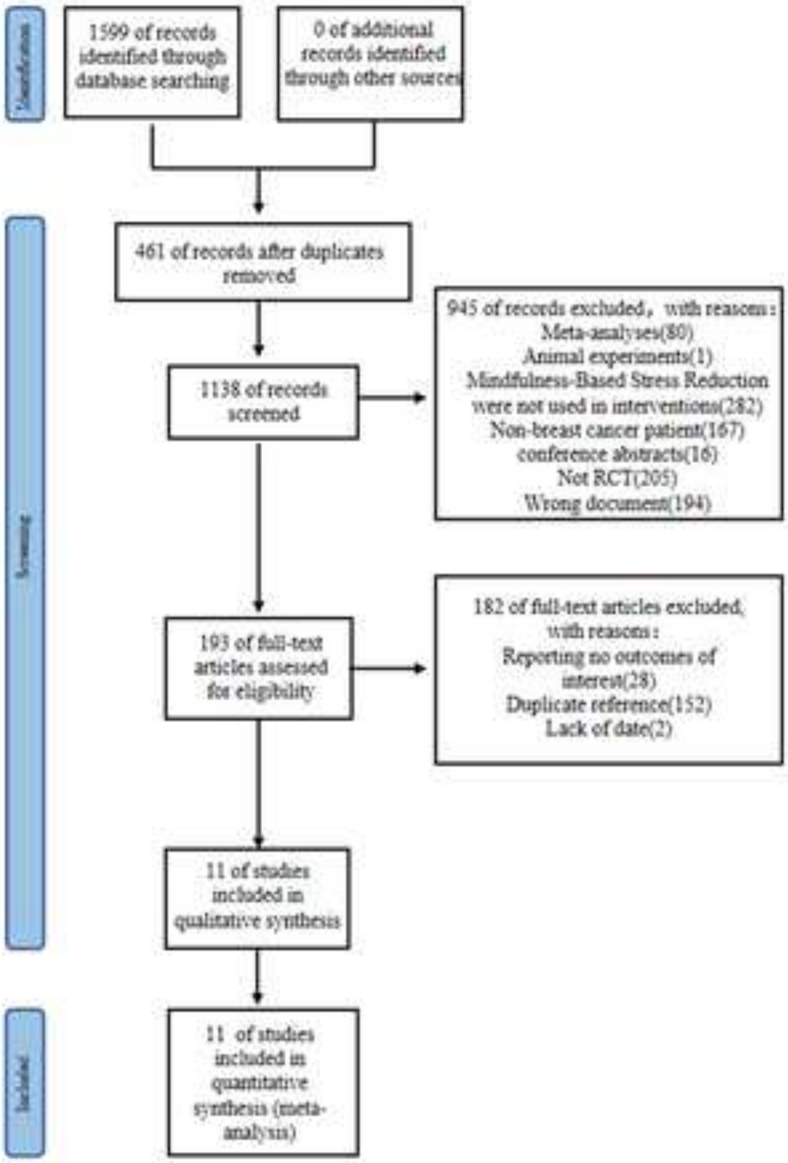

Click here to access/download
Supporting Information
Figure S1.tif

Click here to access/download
Supporting Information
Figure S2.tif

Click here to access/download
Supporting Information
Figure S3.tif

Click here to access/download
Supporting Information
Figure S4.tif

Click here to access/download
Supporting Information
Figure S5.tif

Click here to access/download
Supporting Information
PRISMA-P-checklist.doc
